# Probabilistic projections of baseline twenty-first century CO_2_ emissions using a simple calibrated integrated assessment model

**DOI:** 10.1007/s10584-021-03279-7

**Published:** 2022-02-24

**Authors:** Vivek Srikrishnan, Yawen Guan, Richard S. J. Tol, Klaus Keller

**Affiliations:** 1 Department of Biological & Environmental Engineering, Cornell University, Ithaca, NY USA; 2 Department of Statistics, University of Nebraska, Lincoln, NE USA; 3 Department of Economics, University of Sussex, Sussex, UK; 4 Institute for Environmental Studies, Vrije Universiteit Amsterdam, Amsterdam, The Netherlands; 5 Department of Spatial Economics, Vrije Universiteit Amsterdam, Amsterdam, The Netherlands; 6 Department of Geosciences, Pennsylvania State University, University Park, PA USA; 7 Earth and Environmental Systems Institute, Pennsylvania State University, University Park, PA USA; 8 Thayer School of Engineering, Dartmouth College, Hanover, NH USA

**Keywords:** CO_2_ emissions, Integrated Assessment, Probabilistic Projections, Sensitivity Analysis

## Abstract

Probabilistic projections of baseline (with no additional mitigation policies) future carbon emissions are important for sound climate risk assessments. Deep uncertainty surrounds many drivers of projected emissions. Here, we use a simple integrated assessment model, calibrated to century-scale data and expert assessments of baseline emissions, global economic growth, and population growth, to make probabilistic projections of carbon emissions through 2100. Under a variety of assumptions about fossil fuel resource levels and decarbonization rates, our projections largely agree with several emissions projections under current policy conditions. Our global sensitivity analysis identifies several key economic drivers of uncertainty in future emissions and shows important higher-level interactions between economic and technological parameters, while population uncertainties are less important. Our analysis also projects relatively low global economic growth rates over the remainder of the century. This illustrates the importance of additional research into economic growth dynamics for climate risk assessment, especially if pledged and future climate mitigation policies are weakened or have delayed implementations. These results showcase the power of using a simple, transparent, and calibrated model. While the simple model structure has several advantages, it also creates caveats for our results which are related to important areas for further research.

**Supplementary Information:**

The online version contains supplementary material available at 10.1007/s10584-021-03279-7.

## Introduction

What is a sound approach to projecting future climate change and its impacts? This is a critical question for understanding the impact of adaptation and mitigation strategies. Projected climatic changes hinge on Earth system properties and future drivers, including anthropogenic carbon dioxide (CO_2_) emissions. Projections of future anthropogenic CO_2_ emissions are deeply uncertain; there is no consensus about the probability distribution of future emissions (Ho et al. 2019). Poorly calibrated emissions projections translate into poor climate projections and contribute to poor risk management decisions (Morgan and Keith 2008).

One approach, adopted in the reports of the Intergovernmental Panel on Climate Change, or IPCC (IPCC 2014), to handling the deep uncertainty associated with future emissions and the associated radiative forcing is to use scenarios which cover an appropriate range of plausible futures. For example, the Shared Socioeconomic Pathways, or SSPs (O’Neill et al. 2014; Riahi et al. 2017) explore a plausible range of future emissions through an internally consistent set of future socioeconomic narratives. These scenarios are useful for creating a set of harmonized assumptions for modeling and impacts studies. As they are not intended to be interpreted as predictions of future socioeconomic, emissions, or climate trajectories, they are explicitly provided without probabilities or likelihoods (van Vuuren et al. 2011). This framework is useful for many purposes. However, this lack of probabilistic information makes them difficult to integrate into risk assessment for climate change impacts or adaptation and makes their interpretation susceptible to the cognitive biases that interfere with decision-making under uncertainty (Tversky and Kahneman 1974; Morgan et al. 1992; Webster et al. 2001). For example, one problematic approach is to view all scenarios as equally likely (Wigley and Raper 2001).

Another more recent example is the controversy over presentations of the highest Representative Concentration Pathway, RCP 8.5 (Riahi et al. 2011), as a baseline scenario for impacts (Hausfather and Peters 2020a). We refer a “baseline scenario” as one with no inclusion of the effects of mitigation policies beyond those currently implemented, rather than the original use of the term in the integrated assessment modeling (IAM) literature, where “baseline” refers specifically to a scenario generated by a model run with no forced mitigation through climate policies (van Vuuren et al. 2011). Critics of the interpretation that RCP 8.5 represents a baseline (no inclusion of additional mitigation policies beyond those currently implemented) radiative forcing pathway raise concerns about the increase in coal energy share, relative to present trends, required to achieve this forcing by IAMs (Ritchie and Dowlatabadi 2017a; 2017b; Hausfather and Peters 2020a; Burgess et al. 2021). Indeed, as noted by Hausfather and Peters (2020a) and Skea et al. (2021), current International Energy Agency (IEA) projections diverge from the emissions required by higher-emitting scenarios such as RCP 8.5, and O’Neill et al. (2020) list keeping scenarios up to date and adding additional scenarios in more risk-relevant areas of the scenario space as two key future needs for scenario development. On the other hand, Schwalm et al. (2020) observes that the emissions trajectory associated with the older standalone RCP 8.5 (rather than the newer joint SSP5-8.5 scenario (Kriegler et al. 2014)) closely tracks recent emissions, though this arises from differences between projected and observed land-use change emissions rather than fossil emissions (Hausfather and Peters 2020b). Additionally, along with RCP 4.5, RCP 8.5 bounds a reasonable range surrounding projected emissions through 2050, particularly when land-use change emissions are included. As a result, the authors conclude that RCP 8.5 continues to provide value as a short-to-medium term mean scenario and a longer-term tail-risk scenario.

Both of these seemingly antagonistic perspectives are driven and justified by different use cases and information needs. In this case, the epistemic value provided by RCP 8.5 depends on the question posed and whether the goal is to identify a range of illustrative outcomes or to enumerate probabilities of outcomes to assess and manage risk. This latter consideration is complicated by the underlying deep uncertainties. For example, there is little consensus on key drivers such as projections of future economic growth (Christensen et al. 2018) or the penetration rates of zero-carbon technologies in the global energy mix. Uncertainty about the future strength and direction of mitigation policies further complicates the calculation of probabilities. The potential role of negative emissions technologies (NETs) is another deep uncertainty that directly affects projected emissions trajectories. These considerations demonstrate the need for a transparent and systematic understanding of the relevant assumptions and dynamics and underpin a particular probabilistic projection, so analysts and decision-makers can better understand and account for caveats associated with the resulting probabilities.

A key consideration in producing probabilistic projections is the trade-off between two competing modeling objectives: realistic dynamics to capture key processes and utilize interpretable parameters versus sufficiently fast model evaluations to enable careful uncertainty characterization and quantification. The previous literature on probabilistic emissions projections demonstrates a variety of approaches to navigating this trade-off, which are motivated by the underlying research question. When highly detailed, computationally expensive “bottom-up” models are used, such as the Global Change Assessment Model (GCAM) or the Emissions Prediction and Policy Analysis model (EPPA), one option is to focus on the influence of a few uncertain inputs, as this uncertainty space can be captured with a relatively small-to-moderate number of samples (e.g., Capellán-Pérez et al. 2016; Fyke and Matthews 2015). One downside to this approach with complex models is that the impact of interactions between the inputs treated as uncertain and those which are not may be missed. van Vuuren et al. (2008) use a broader set of inputs, but focus on sampling around each of the no-policy IPCC-SRES scenarios rather than sampling the entire probability space. A larger set of samples can be used with an emulator of the full model (e.g., Webster et al. 2002; Sokolov et al. 2009; Webster et al. 2012; Gillingham et al. 2018), as emulation allows for many more model evaluations within a fixed computational budget, resulting in an ability to explore many more sources of uncertainty at the expense of losing some of the dynamical richness of the full model (depending on the type of emulator or response surface used). Gillingham et al. (2018) go further in building emulators of multiple IAMs, which are used to understand the impact of model structural uncertainty on the resulting projections. Another approach is the use of a Bayesian statistical model calibrated using historical data (e.g., Raftery et al. 2017; Liu and Raftery 2021). These models can also be run many times, allowing them to fully resolve the tails of the projective distributions, and are flexible enough to capture historical dynamics while representing different future scenarios and potential trend breaks. However, they may have parameters which are less interpretable, potentially resulting in fewer insights from examining sensitivities and marginal parameter distributions.

This spread of approaches is valuable as it reveals the impacts of the underlying modeling assumptions and included uncertainties on the resulting projections. In this study, we add to this literature by using a simple, mechanistically motivated integrated assessment model. The simplicity of the model permits millions of model evaluations without requiring a reduced-form emulator, allowing full statistical calibration of the model on historical data and insights into the dynamics of the full model. This level of simplicity provides epistemic benefits by making full uncertainty quantification computationally tractable (Helgeson et al. 2021), much like the statistical approach of Raftery et al. (2017) and Liu and Raftery (2021). The mechanistically motivated model structure allows us to incorporate theoretical insights about the dynamics and structure of the relationships between interpretable model parameters using prior distributions drawn from the literature.

We focus on projecting baseline CO_2_ emissions for the remainder of the twenty-first century, only incorporating the effects of those mitigation policies which have had sufficient effect to be reflected in the calibration data (both historical observations and expert assessments made under the baseline assumption). These baseline emissions projections will not necessarily be consistent with the 2010 baselines adopted by the SSPs, as we calibrate, constrain, and initialize our model using more recent observations which have been influenced by past and current policies, as well as expectations about potential future policies. We also neglect the impact of currently unproven but potentially impactful NETs, as it is unclear what technologies might eventually penetrate on a wide scale, when they might do so, and what their level of negative emissions may be (Fuss et al. 2014; Smith et al. 2015; Vaughan and Gough 2016). It is also unclear as to whether NETs would be able to achieve a critical deployment level in the absence of additional climate policies (Honegger and Reiner 2018), which we do not consider. As a result, these projections are best understood as a reflection of what CO_2_ emissions might look like without significant changes in mitigation policy implementation or technological development and deployment patterns. We refrain from projecting global mean temperatures, as we do not consider the effects of carbon-cycle and biogeochemical dynamics and their uncertainties, which can have a large impact on the resulting CO_2_ concentrations (Booth et al. 2017; Quilcaille et al. 2018), as well as uncertainties related to climate sensitivity (Goodwin and Cael 2021).

Naturally, the simple structure of our model does mean that some assumptions about the structure are particularly influential, which creates a number of caveats. This analysis illustrates some of the challenges faced in navigating the simplicity-realism trade-off, while also showing the potential for what can be learned using this approach.

## Modeling overview

In this section, we provide a brief overview of the structure of our model. Full details are available in Section S1 of Online Resource 1. As a starting point, we adopt the overall structure of the DICE model (Nordhaus and Sztorc 2013; Nordhaus 2017). While our model structure is similar to DICE, our analysis differs from one of the typical uses of that model (e.g., Nordhaus 1992) as we do not optimize over the space of abatement policies. We expand on the DICE model structure by allowing population growth to be endogenous and affected by economic growth. We also use a different approach to represent changes in the emissions intensity of the global economy, which in our model is the result of successive penetrations of technologies with varying emissions intensities. The resulting model structure involves a logistic population growth component with an uncertain saturation level. We model global economic output using a Cobb-Douglas production function in a Solow-Swan model of economic growth. Population and economic output influence each other through changes in per-capita consumption and labor inputs.

Economic output is translated into emissions using a mixture of four emitting technologies: a zero-carbon pre-industrial technology, a high-carbon intensity fossil fuel technology (representative of coal), a lower-carbon intensity fossil fuel technology (representative of oil and gas), and a zero-carbon advanced technology (representative of renewables and nuclear). The lack of NETs in our modeling framework means that we cannot fully explore the bottom of the emissions range captured by the SSP-RCP framework, as several of these scenarios, namely SSP1-1.9, SSP2-2.6, and SSP4-3.4, include their penetration prior to 2100.

We treat all model parameters, including emissions technology penetration dynamics, as uncertain. The statistical model accounts for cross-correlations across the model errors of the three modules as well as independent observation errors. We calibrate the model using century-scale observations of population and global domestic products per capita (Bolt and van Zanden 2020) and anthropogenic CO_2_ emissions (excluding land use emissions) (Boden et al. 2017; Friedlingstein et al. 2020). As the Bolt and van Zanden (2020) data extend only to 2018, we extend them to 2020 using World Bank data (The World Bank 2020) for global domestic product per capita and United Nations data for population (United Nations, Department of Economic and Social Affairs, Population Division 2019). We also probabilistically invert (Kraan and Cooke 2000; Fuller et al. 2017) three expert assessments in our calibration procedure to gain additional information about prior distributions and potential future changes to population (United Nations, Department of Economic and Social Affairs, Population Division 2019), economic output (Christensen et al. 2018), and CO_2_ emissions (Ho et al. 2019) which are not reflected in the historical data. More information about the model calibration procedure is available in Section S2 of Online Resource 1, while full details on the derivation of the likelihood function and the choice of prior distributions are available in Sections S3 and S4 of Online Resource 1, respectively.

Two key deep uncertainties affecting probabilistic projections of CO_2_ emissions are (i) the size of the fossil fuel resource base (Capellán-Pérez et al. 2016; Wang et al. 2017) and (ii) the prior beliefs about the penetration rate of zero- or low-carbon energy technologies (Gambhir et al. 2017). First, we consider the impact of unknown quantities of remaining fossil fuel resources, which are fossil fuel deposits which are potentially recoverable (as opposed to reserves, which are available for profitable extraction with current prices and technologies) (Rogner 1997). Estimates of the fossil fuel resource base vary widely (McGlade 2012; McGlade et al. 2013; Mohr et al. 2015; Ritchie and Dowlatabadi 2017a). Recent criticisms of the continued use of high-emissions and high-forcings scenarios focus on the plausibility of the required amount of fossil fuels, particularly coal, required to generate the emissions associated with these scenarios (Ritchie and Dowlatabadi 2017a; 2017b). For the penetration rate of low-carbon technologies, historical emissions data can only provide limited information about this transition due to the relatively limited penetration of these technologies to date and differences in the penetration dynamics of other generating technologies such as coal, oil, and natural gas (Gambhir et al. 2017).

We focus on these deep uncertainties to illustrate the sensitivity of probabilistic projections of emissions, and therefore temperature anomalies, to these assumptions, while recognizing that there are other influential deep uncertainties. To account for the impact of deep uncertainties and to simplify the discussion, we design four scenarios. Our “standard” scenario assumes a fossil fuel resource base consistent with the best guess resource estimates from Mohr et al. (2015) and uses a truncated normal prior distribution for the half-saturation year of zero-carbon technologies (that is, the year when that technology achieves a 50% share of the energy mix). This distribution assigns a 2.5% prior probability to half-saturation between 2020 and 2050, and has its mode at 2100. Our “low fossil-fuel” and “high fossil-fuel” scenarios use the same prior distribution over the zero-carbon technology half-saturation year, but adopt the low and high resources estimates, respectively, from Mohr et al. (2015). These three scenarios allow us to explore the implications of varying assumptions about fossil fuel supplies, with the associated knock-on effects for energy-generating costs, on CO_2_ emissions.

We also examine the sensitivity of our projections to a more pessimistic set of prior beliefs about zero-carbon technology penetration. In this “delayed zero-carbon” scenario, we assign only a 2.5% prior probability to global half-saturation by 2100. To isolate the impact of this prior distribution, we use the same fossil fuel resource constraint as in the standard scenario.

In each of these scenarios, if a set of parameters results in fossil-fuel consumption exceeding the size of the resource base for any fuel, it is excluded from the calibration set. This induces a trade-off between economic growth and fossil-fuel consumption, such that economic growth is slowed down if the economy is still dependent on limited fossil-fuel resources. While we do not explicitly model the prices of fossil fuels, this resulting effect is analogous to the effect of increasing prices due to relative resource scarcity. However, for a fixed rate of economic growth, technology succession can occur earlier than the latest year associated with the fossil fuel constraint, representing changing demand as the driver of technological change, rather than restrictions in supply.

By itself, this fossil-fuel constraint does not rule out ahistorical fuel substitution dynamics despite the statistical calibration procedure, as uncertainties in and correlations between the emissions intensities of the fossil-fuel technologies, the half-saturation years of the various technologies, and the rate of technological penetration create large degrees of freedom in mapping historical economic growth to CO_2_ emissions. For example, some parameter sets might imply a 50% zero-carbon share and/or a less than 10% coal share in 2020 (see Fig. S1 in Online Resource 1). To constrain this behavior, we add a further constraint on technology shares in 2019. Based on fuel share data from the last 10 years (BP 2020), we require that the coal share in 2019 is between 20 and 30% and that the zero-carbon share is between 10 and 20%. These windows were chosen to be relatively wide to acknowledge year-to-year volatility in these shares. This constraint captures one of the most notable differences between our “baseline” scenarios and the no-policy “baseline” SSP-RCP scenarios, as the share of these various technologies has been influenced by past and current policies such as subsidies and tax credits.

**Fig. 1:**
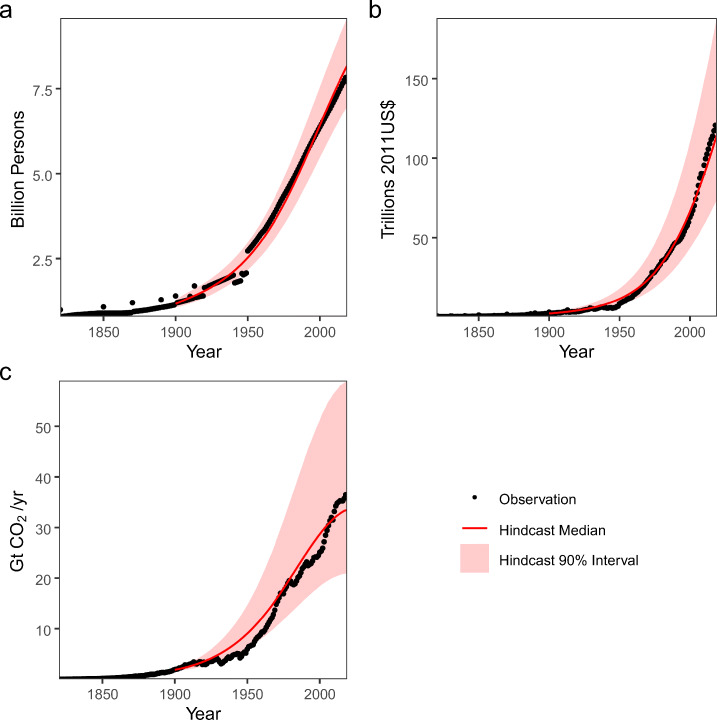
Model hindcast results across all model outputs – Median and 90% credible intervals for model hindcasts from 1900 to 2019 conditioned on data from 1820 to 1899. Hindcasts are provided for all three model components: (a) global population, (b) gross world product, and (c) global CO_2_ emissions

Our model simulations also include error terms to account for discrepancies between the data and the model outputs (Brynjarsdóttir and O’Hagan 2014). These error terms are generated conditionally using the model discrepancy process (see Section S3 of Online Resource 1) conditional on the discrepancies over a period of years which are selected to be fixed. For example, the discrepancy terms for projections are sampled conditionally on the model-data discrepancies over the 1820–2019 historical period to preserve the calibrated variance structure of the discrepancies.

## Calibration results

We conduct a hindcasting exercise and perform cross-validation to assess potential biases of the calibrated model and to evaluate out-of-sample projection skill. For the hindcast, we make projections for the period from 1900 to 2019 conditional on data from 1820 to 1899 (see Fig. 1). The median growth simulations slightly underestimate post-2000 economic growth. As a result, the model was unable to reproduce the rapid growth in emissions starting around 2000, while it did capture the recent slowing of the emissions growth rate. The hindcasts also demonstrate a large degree of underconfidence in the post-1950 period.

We further test the model predictive skill use a *k*-fold cross-validation procedure. We randomly sample fifty hold-out test data sets (each corresponding to 40 years). The model is re-calibrated with the remaining training data, and we generate simulated data for the held-out years conditional on the training data. The average cross-validation coverage of the 90% credible intervals for the held-out data are 92% for population and economic output and 93% for emissions, suggesting good out-of-sample predictive performance despite the hindcast’s underconfidence. The underconfidence seen in the hindcast is likely the result of the accumulation of errors over the 120-year hindcasting period. These errors can grow quite large, as the model’s projections and the data do not diverge strongly until later in the twentieth century across all parameter vectors in the calibrated parameter set. However, we note that this level of underconfidence is similar to that seen in Raftery et al. (2017) despite the many differences between the two models, suggesting that this may be a consequence of using a statistically calibrated, relatively simple model.

## Projections of future CO_2_ emissions

**Fig. 2:**
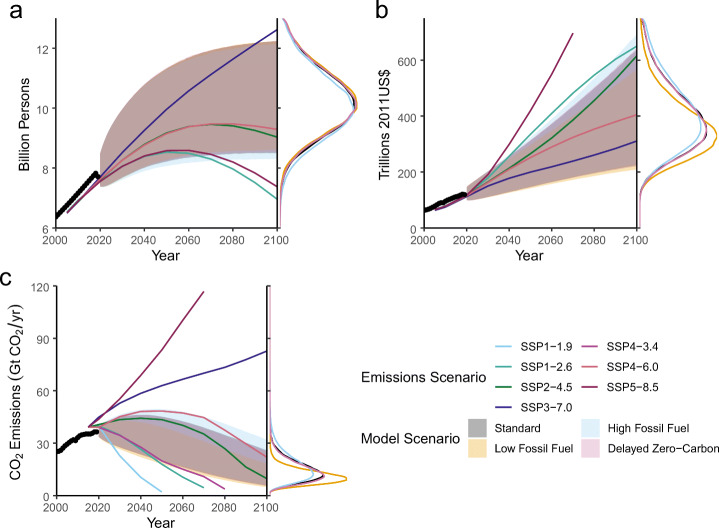
Projections of model outputs – Projections of (a) global population (billions of persons), (b) gross world product (trillions 2011US$), and (c) annual carbon dioxide emissions projections from 2020 to 2100 for the considered scenarios. The shaded regions are the central 90% credible intervals. Black dots represent observations from 2000 to 2019. The marginal distributions of each output in 2100 are shown on the right. The relevant quantities from the marker baseline SSP-RCP emissions scenarios (Riahi et al. 2017; Rogelj et al. 2018; O’Neill et al. 2016) are shown for comparison by the lines

Deep uncertainty about fossil fuel resources causes more variability in CO_2_ projections than prior beliefs about zero-carbon penetration (Fig. 2c). Under the base scenario assumptions, the median cumulative emissions from 2020 to 2100 are 2200 GtCO_2_, with the 90% prediction interval covering 1500–3000 GtCO_2_. The lower end of the prediction interval, as well as the median levels, are only slightly influenced by the fossil fuel constraint, varying by 100–200 GtCO_2_ at most, which is expected as the constraint does not affect simulations with faster decarbonization than is required by the supply-side limit. On the other hand, the supply constraint matters more at the upper end, as the low-fossil fuel scenario prediction interval’s upper bound is 2600 GtCO_2_, with the high-fossil fuel interval extending to 3400 GtCO_2_. This is largely the result of varying the coal constraint, as residual coal emissions can get close to the resource limit when the penetration of lower- and zero-emitting technologies is slower and economic growth rates are more rapid. This is consistent with the analysis by Capellán-Pérez et al. (2016), conducted using the more detailed GCAM, which found that, when considering resource constraints, cumulative CO_2_ emissions was mainly sensitive to the size of the coal resource base. Both of these results further agree with the argument by Ritchie and Dowlatabadi (2017a) and Ritchie and Dowlatabadi (2017b) that the relative plausibility of higher-emissions scenarios is dependent on their assumptions about coal resource availability and utilization.

The main impact of the technology penetration constraint is to sharply constrain the half-saturation years of the various generating technologies (*τ*_*i*_ in Fig. S2), and, due to correlations, the inferred emissions intensity of the higher-emissions technology (*ρ*_2_ in Fig. S2). Due to this sharpening of the half-saturation year of the zero-emissions technology (*τ*_4_ in Fig. S2), there is little difference between the standard and delayed zero-carbon scenario penetrations, despite the influence of the CO_2_ emissions expert assessment from Ho et al. (2019) in the calibration (see Fig. S3 and Fig. S4; including the CO_2_ expert assessment increases the upper tail areas of the non-low fossil fuel scenarios, but does not result in a separation of the standard and delayed zero-carbon scenarios despite their different priors, as the marginal distributions of the emissions parameters are the same regardless of what assessments are used). The impact of the constraint on emissions projections can be seen in Fig. S5. The technological constraint also induces a typical half-saturation year in the second-half of the twenty-first century, with the 90% central credible interval between 2057 and 2086, and a median half-saturation year of 2071 (see Fig. S6 for how the distribution of shares of our technologies change over the remainder of the century). These quantities are relatively insensitive to the resource constraint.

After accounting for the additional emissions from 2015 to 2019, the base median estimate of 2100 GtCO_2_ is slightly higher than that from the “Continued” forecast from Liu and Raftery (2021), which had a median cumulative emissions from 2015 to 2100 of 2100 GtCO_2_. It should be noted that this Liu and Raftery (2021) scenario assumes that nations will meet their Nationally Determined Contributions (NDCs), and therefore assumes additional mitigation policies beyond those captured by our model’s data. The relatively small difference is likely due to the logistic penetration curve in our model, which causes a faster rate of decarbonization after half-saturation compared to the constant rate of post-NDC carbon intensity decrease in the Liu and Raftery (2021) scenario. However, all of our projections are much lower than the base forecasts by Liu and Raftery (2021), which could be due to their spatial disaggregation, as economic growth with slower decarbonization can result in lower cumulative emissions from countries and regions with relatively small economies and, therefore, low levels of emissions despite high projected utilizations of fossil fuels. In our model, the global economy decarbonizes uniformly, the rate of which (due to the penetration constraint) is heavily influenced by the energy mix in developed countries. This suggests that our projections may be biased towards lower levels of emissions by the level of aggregation, as globally aggregated projections can mask the importance of regional deviations from the global trend (Pretis and Roser 2017).

Our model’s projections are broadly consistent with the extrapolations from current International Energy Agency (IEA) projections made by Hausfather and Peters (2020a). Indeed, in the IEA Stated Policies scenario (IEA 2020), emissions in 2030 are 36 GtCO_2_/year, which is the median value of our high fossil fuel scenario (90% predictive interval of 28–46 GtCO_2_/year) and close to the median (35 GtCO_2_/year in 2030) of our standard scenario’s projections (90% predictive interval 28–45 GtCO_2_/year) and low-fossil fuel projections (median 34 GtCO_2_/year, 90% predictive interval 27–43 GtCO_2_/year). The IEA’s Sustainable Development scenario, which projects 27 GtCO_2_/year, is just in the lower tail of most of our projections. Unsurprisingly, then, the bulk of our predictive distributions, as shown in Fig. 2, correspond to the range deemed by Hausfather and Peters (2020a) to be “likely” given current policies. However, as with the Hausfather and Peters (2020a) analysis, our model does not account for land-use emissions, which could increase emissions sufficiently to be more consistent with higher-emitting SSP-RCP scenarios (Schwalm et al. 2020). Furthermore, our model cannot by its structure account for the possibility of technological backsliding, which would result in increase in global emissions intensities due to, e.g., an increase in coal use in varying regions around the world or the replacement of nuclear plants with fossil-fueled generation. These possible trend breaks are also not reflected in projections such as those from the IEA, which are mainly based on extrapolating current trends (Skea et al. 2021). For example, as noted by Skea et al. (2021), this class of projections anticipates nuclear generation growth by 39% on average.

**Fig. 3:**
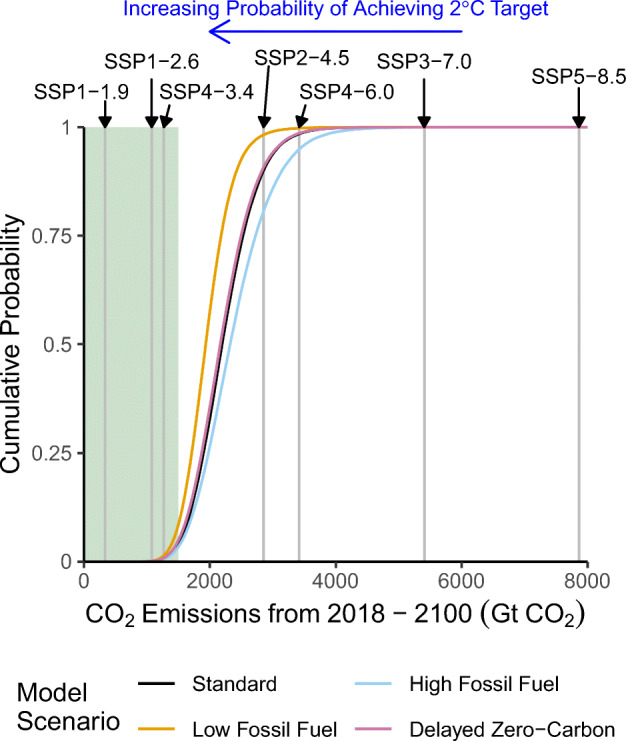
Cumulative emissions projections from 2018 to 2100 – Cumulative density functions for cumulative emissions projections for the four model scenarios. The grey lines are the cumulative emissions over this period for the labelled IPCC scenario. The green region represents cumulative emissions which are consistent with at least a 50% probability of achieving the 2^∘^C Paris Accords target (Rogelj et al. 2018)

The projections for the remainder of the century broadly span the area captured by those SSP-RCP scenarios which include additional decarbonization, but no NET penetration (see Figs. 2 and 3. In particular, SSP2-4.5 closely tracks the upper range of the standard and delayed zero-carbon scenarios until about 2070, when decarbonization occurs more rapidly in that SSP-RCP scenario than in our projections. SSP4-6.0, on the other hand, is a tail scenario under these assumptions, but characterizes the upper level of the likely range of the high-fossil fuel scenario. The no-policy SSP5-8.5 and SSP3-7.0 scenarios are well outside of our distributions, but for different reasons. Our model considers SSP5-8.5 implausible due to the runaway levels of economic growth (see Fig. 2b) fueled by the wide utilization of extremely large coal reserves. On the other hand, SSP3-7.0 has slower economic growth, but both large population growth (Fig. 2a) and continued fossil fuel use.

The impact of projected economic growth is crucial for interpreting these projections as well-calibrated or biased-downward. Due to our choice of calibration period, our projected rates of per-capita economic growth from 2018 to 2100 are lower than 2% per year (see Fig. S7). This is much lower than the expert assessment reported in Christensen et al. (2018), which was used in both this analysis and Gillingham et al. (2018). With the projected levels of growth from our calibrated model and the rate of decarbonization induced by the penetration constraint, almost all of our simulations show the rate of carbon intensity decreasing more rapidly than the increase in per-capita economic growth. The difference between these two Kaya identity components is greater than in the forecast by Liu and Raftery (2021), which could be the result of our spatial aggregation, as discussed earlier.

Our base scenario simulations contain a small fraction (4%) of outcomes where emissions are reduced rapidly enough to be consistent with at least a 50% probability of achieving the 2^∘^C Paris Agreement target(Rogelj et al. 2018), though we note that this is only in reference to CO_2_ emissions, not the CO_2_ equivalent of emissions of other greenhouse gases, and that we also neglect the impact of emissions from land-use change. We classify these simulations using a classification tree (Breiman et al. 1984; Therneau and Atkinson 2019). These states of the world are characterized by combinations of the rate of technological penetration and the emissions intensity of the lower-emitting fossil technology, rather than the half-saturation year of the zero-carbon technology. In particular, this outcomes only occurs in the simulations if the emissions intensity of the lower-emitting fossil technology is below the 44th percentile of the marginal posterior (see Fig. S2 and S8; *ρ*_3_). If the rate of technological penetration is sufficiently slow (4%/year, which is the 88th percentile of the marginal posterior; see Fig. S2 and S8; *κ*), the emissions intensity of the lower-emitting fossil technology must be no greater than its 32nd percentile (approximately 0.044 GtCO_2_/US$2011). On the other hand, there are no such combinations which characterize relatively high-emissions outcomes, which is likely due to the complex interactions driving rapid economic growth as well as the impact at the high end of residual emissions from coal utilization and the resource constraint.

**Fig. 4:**
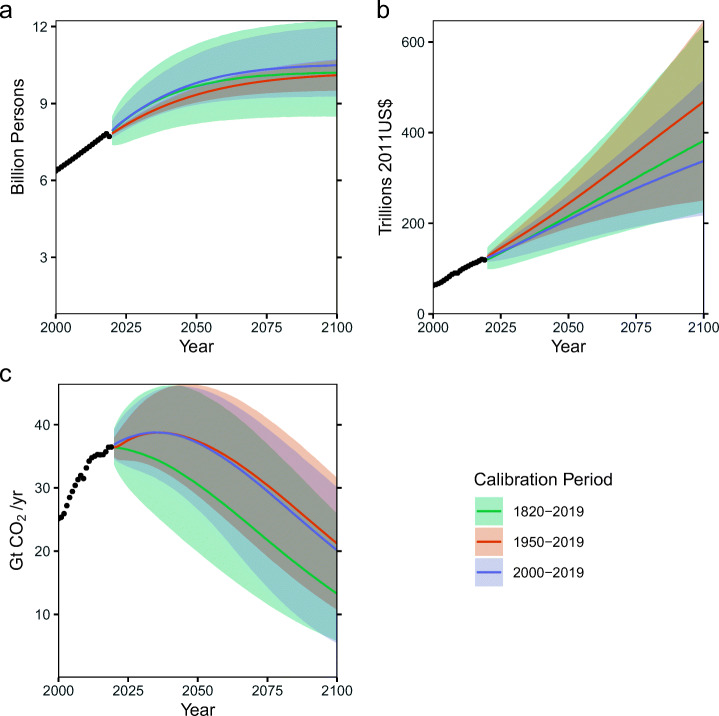
Model projections using different calibration periods – Model projections based on calibrations using data from 1820 to 2019, 1950–2910, and 2000–2019 for (a) global population, (b) gross world product, and (c) global CO_2_ emissions. For each calibration period, the line is the median of the projections, and the ribbon is the 90% credible interval. These projections were made using the “standard” scenario assumptions about fossil fuel resource limits and zero-carbon technology half-saturation year priors

These results depend on our choice of century-scale data (Fig. 4). Using data from 1950 results in higher projected economic growth and emissions by the end of the century despite a reduced overall level of uncertainty due to the growth in both economic output and production after World War II and the exclusion of previous boom-bust cycles, recessions, and depressions. On the other hand, the high-economic growth tail from the 1820–2019 and 1950–2019 calibrations is not present in the 2000–2019 calibration due to the exclusion of the economic growth second half of the twentieth century. This has interesting implications, as even the other calibrations did not result in particularly rapid projected economic growth for the remainder of the twenty-first century. The use of century-scale data also results in lower emissions projections than the use of data starting in the twentieth century due to differences across all of the emissions parameters (see Fig. S7). Using century-scale calibration data results in a higher inferred technological penetration rate, as well as more constrained half-saturation estimates for the fossil technologies. The end result is that the use of shorter data sets produces projections in which emissions peak later in the century, resulting in higher emissions even when economic growth is slower.

## Cumulative emissions sensitivities

**Fig. 5:**
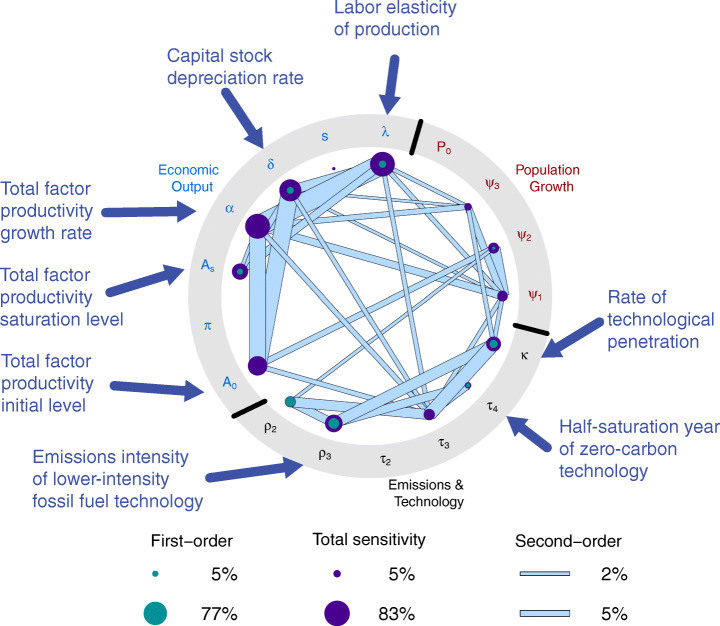
Global sensitivities of cumulative emissions to model variables – Global sensitivity (Sobol’ 1993; 2001) indices for the decomposition of variance of cumulative emissions under our standard scenario from 2018 to 2100. The computation of sensitivity index estimates is described in the Section S4 of Online Resource 1. Filled green nodes represent first-order sensitivity indices, filled purple nodes represent total-order sensitivity indices, and filled blue bars represent second-order sensitivity indices for the interactions between parameter pairs. Important parameters are labelled with their role in the model. Other model variable names are defined in Table S1. Sensitivity values exceeding thresholds are provided in Tables S4 and S5

Cumulative emissions variability is driven mainly by uncertainties in interacting economic and technology dynamics, with a much smaller contribution from population dynamics (Fig. 5). Cumulative emissions exhibit statistically significant sensitivities (in the sense that the 95% confidence interval of the sensitivity index does not contain zero) to all model parameters other than the initial population (*P*_0_), the half-saturation year of the more-intensive fossil fuel technology (*τ*_2_), and the labor force participation rate (*π*). This illustrates a challenge of constructing parsimonious models for projecting emissions. The first- and higher-order sensitivities to a large number of parameters illustrates the complexity of the system dynamics, even for this highly aggregated, relatively simple IAM. Uncertainties in several economic variables, including those characterizing total factor productivity growth, explain a large fraction of the variability in cumulative emissions, showing the importance of improving our understanding of economic growth dynamics in addition to technological shifts in the energy sector if we are to further constrain future emissions projections.

Economic variables matter more through higher-order sensitivities and interactions with other parameters, while variables related to emissions intensities and technological substitutions have a more direct influence. This is due to the translation of economic growth into emissions through the mixture of emitting technologies within the model. While it may be surprising that the half-saturation year of the zero-carbon technology (*τ*_4_) has a relatively small influence, this results from the same model dynamics discussed earlier when characterizing low-emissions tail scenarios. As the marginal posterior of *τ*_4_ is relatively narrow and is limited to the second-half of the twenty-first century, the rate of technological penetration (*κ*, which influences the shape of the logistic curves) and the emissions intensities of the fossil technologies (*ρ*_2_ and *ρ*_3_) explain most of the uncertainty in mapping economic output to emissions. These large sensitivities highlight the need for updated accounting of emissions factors to help constrain and update projections of CO_2_ emissions. The emissions intensity then combines with economic growth, which is dominated by total factor productivity, or TFP (as discussed by Nordhaus (2008) and Gillingham et al. (2018)), as well as the dynamics of capital, including depreciation (controlled by the capital depreciation rate *δ* and the capital elasticity of production (1 − *λ*, where *λ* is the labor elasticity).

It is worth discussing further the importance of TFP growth in this context. The growth rate (*α*) explains a large degree of cumulative emissions variability through its intersection with other parameters, even though it has a non-statistically sensitivity first-order sensitivity. In this model, there is a strong interaction between the TFP growth rate and the elasticity of production with respect to capital, as these directly affect production growth. Future TFP growth dynamics are likely to be nonstationary compared to our inferences from the historical record, due to the increasing penetration of automation in the global economy, though a more detailed representation of these dynamics might also include trend breaks in TFP growth and an accounting of the displacement effect of automation on the labor share (Acemoglu and Restrepo 2019). Our model’s lack of ability to account for accelerating TFP growth, and therefore even faster economic growth, could partially explain our lower growth projections compared to Christensen et al. (2018), as well as the lack of higher emissions scenarios in our projections.

We do not observe large sensitivities to population dynamics. This is consistent with the analyses from Raftery et al. (2017), which found only a 2% sensitivity of CO_2_ emissions in 2100 to population, as well as Gillingham et al. (2018), which found a 10% sensitivity. This could be the result of our model’s global aggregation, as some regionally focused analyses have found that population growth is the Kaya identity component most typically associated with changes in CO_2_ emissions (e.g., van Ruijven et al. 2016). However, our analysis does contradict the finding of van Vuuren et al. (2008), which identitifed population as a major driver of CO_2_ emissions.

## Discussion

In this analysis, we produced baseline probabilistic CO_2_ projections from a simple, mechanistically motivated integrated assessment model calibrated on century-scale historical data under several realizations of different deep uncertainties. This type of modeling exercise has several potential virtues. Our model runs rapidly enough to be statistically calibrated using a long data set as well as to be subjected to a global sensitivity analysis. The coupled uncertainty- and sensitivity- analyses allow us to identify important linkages across different modeling components and interpretable model parameters, illustrating the complexity of the joint social-economic-technical system which ultimately results in CO_2_ emissions. Some of these linkages would not be directly seen when using a more computationally expensive model which might preclude the required number of model evaluations. By comparing the resulting probabilities across the different scenarios corresponding to the considered deep uncertainty, we could explore the impacts of varying assumptions. For example, we could see the impact of fossil fuel resource uncertainty on the high-emissions upper tail.

One clear lesson is that even for this relatively stylized and highly aggregated model, calibration using historical data is not itself sufficient to fully constrain the model dynamics. For one, the choice of calibration period matters substantially in projecting economic growth and emissions intensities. Second, even this simple model has enough degrees of freedom to produce inaccurate energy-generating technology shares without strong constraints (which still result in underconfident hindcasts). The imposition of these constraints results in consistency with IEA Stated Policy projections (IEA 2020) through 2030. However, our projections assume that current technological substitution trends will continue or accelerate, and do not account for the possibility of technological backsliding. It would be possible and interesting to use a modeling framework similar to ours to understand the extent to which backsliding and increased economic growth could combine to produce high-emissions outcomes which seem unlikely based on current and historical trends. Our projections are also made under the baseline assumption that no new mitigation policies will be implemented. This assumption is unlikely to be true in practice, particularly as the impacts of climate change become more apparent, and we use it purely as a counterfactual.

Global aggregation also runs the risk of masking local and regional dynamics which could be influential in determining how future emissions change, particularly in regions which have so far either no contributed much to total CO_2_ emissions or which are experiencing rapid economic growth (Pretis and Roser 2017). These could be strong enough to cause trend breaks from the historical dynamics, resulting in emissions higher than those projected by our modeling exercise or the IEA, particularly if currently planned mitigation policies are not fully implemented or are abandoned in the face of political or economic pressures. With its current formulation, our model, and therefore the projections, are incapable of addressing this possibility, which could result in the type of technological backsliding discussed earlier. Our model could, however, be adapted to explore scenarios which allow for non-constant rates of technological penetration or the possibility of an older emitting technology to recapture higher shares of the global energy mix. Another example is the potential impact of population growth in regions such as sub-Saharan Africa, which were a cause of the relative lack of sensitivity of emissions to population growth in Raftery et al. (2017). It would be interesting to use a spatially disaggregated version of our model to explore the combinations of population and economic growth and technological penetrations which would result in increased emissions outside of the likely range reported here.

There are several other caveats that are important to mention. While the economic outputs in our analysis suggest that the higher end of economic growth forecasts, such as those elicited in Christensen et al. (2018), will not be achieved (even before accounting for shocks such as the COVID-19 pandemic), this is partially dependent on both our choice of calibration period as well as the structure of our economic model, which rules out the possibility of trend breaks in growth resulting from, e.g., automation. Additionally, our study is silent on the impacts of negative emissions technologies. Changes in climate policies could result in these technologies becoming viable and widespread prior to the end of the century. This would shift emissions downward starting from the point when these technologies begin to penetration. This effect depends, however, on the currently uncertain details of these technologies. One extension of this study might be to include these deep uncertainties, producing projections which are conditional on both the negative emissions technologies and the rate of penetration of a sample technology. Finally, while baseline scenarios are a useful counterfactual, climate policies evolve, and the odds of policies remaining the same through 2100 are nearly zero. The resulting changes in incentives for technology and energy-use would necessarily alter the dynamics captured by our model calibration.

It is important to stress that our analysis has also neglected the large effect of the Earth-system response to changes in CO_2_(Friedlingstein et al. 2014; Booth et al. 2017; Quilcaille et al. 2018). From the perspective of managing climate risk, emissions projections and forecasts should be understand in the context of these large climate-system uncertainties. It would be unwise to ignore the implications of higher-emissions scenarios for risk analysis by focusing only on their emissions trajectories, as higher levels of radiative forcing and changes to global mean temperature could be obtained from lower emissions levels than those used in scenario generation. Moreover, these scenarios have important value in climate modeling analyses due to their high signal-to-noise ratio. Our analysis here is not intended to downplay the value of these scenarios for understanding the climate system or for climate risk analysis. We also did not model non-CO_2_ greenhouse gas emissions.

Ultimately, even with these caveats, simple, mechanistically motivated models have a role to play in understanding the uncertainties associated with future climate risks. Many of the limitations discussed above could be addressed by transparently creating or modifying such a model and comparing projections, parameter distributions, and sensitivities across assumptions. The systems which produce anthropogenic CO_2_ are sufficiently complex that the ability to map out which parts of the system interact with other parts to produce higher- or lower emissions is important to improve our understanding of climate risk. More computationally expensive and complex models are valuable in representing these complex system dynamics from the ground up, but may not be amenable to this type of analysis without the use of emulators, which smooth out the model dynamics to some degree and may restrict the number of parameters which can be considered (depending on the type of emulator). Ultimately, all models are wrong (Box 1976), but the use of multiple models with varying levels of complexity and which transparently make different assumptions can help us gain a holistic view of future climate risk and its drivers.

## Supplementary Information

ESM 1 (PDF 3.88 MB)
