# Supplementary material for: Probabilistic projections of baseline twenty-first century CO2 emissions using a simple calibrated integrated assessment model
Source: Clim Change. 2022 Feb 24;170(3-4):37. doi: 10.1007/s10584-021-03279-7 (PMC8866549; doi:10.1007/s10584-021-03279-7)
Supplement: Supplementary file 1 — (PDF 3.88 MB) [file 10584_2021_3279_MOESM1_ESM.pdf]

Highest CO2 Emissions Scenarios Are Extreme Given Observations and Expert Judgements

Supplemental Materials

Vivek Srikrishnan Yawen Guan Richard S. J. Tol
Klaus Keller

S1 Model Structure

Model outputs are globally-aggregated population (in billions), gross world product (in trillions 2011 US$), and carbon emissions (in GtC/yr), with an annual temporal resolution. The model generates these outputs using a set of three coupled modules: (1) population; (2) economic output; (3) carbon emissions. We could have followed the Kaya Identity and included energy use. However, CO_2 emissions are not measured but instead imputed from energy use. Including energy use would not add information, but it would increase the dimensionality of the model.

There is a bidirectional coupling between population and economic output: population affects labor inputs through the labor force participation rate, while per-capita income affects the rate of population growth. CO_2 emissions are a consequence of economic output through a mixture of emitting technologies with varying emissions intensities.

Population

We model population growth using a logistic model (Cohen, 1995), modified by an income-sensitive growth rate. At time t, population P_t is given by

$$P_t = P_{t-1}[1 + \psi_1(y_{t-1}/(\psi_2 + y_{t-1}))((\psi_3 - P_{t-1})/\psi_3)],$$

where y is annual per-capita income, ψ_1 is the annual population growth rate, ψ_2 is the half-saturation parameters with respect to per-capita consumption, and ψ_3 is the logistic carrying capacity. This model structure allows for interactions between per-capita income and population growth. Note that this equation implies that the population grows before stabilizing near saturation; other projections have been made which assume population peaks and then decreases (Lutz et al., 2001).

Economic Output

To model gross world product, we use a Cobb-Douglas production function in a Solow-Swan model of economic growth. Total world production Q_t at time t is

$$Q_t = A_t L_t^{\lambda} K_t^{1-\lambda},$$

where A is total factor productivity, L is labor input, K is capital input, and λ is the elasticity of production with respect to labor. Each year, total production is divided between consumption and investment,

$$Q_t = U_t + I_t = (1 - s)Q_t + sQ_t,$$

where s is the savings rate, which we assume to be constant.

Growth in total factor productivity (TFP) occurs exogenously. The dynamics of long-term technological change are deeply uncertain(Starr and Rudman, 1973; Ausubel, 1995). Following Nordhaus and Yohe (1983), we allow TFP to saturate as the population ages and becomes less innovative:

$$A_t = A_{t-1} + \alpha A_{t-1}\left[1 - \left(\frac{A_{t-1}}{A_s}\right)\right],$$

where α is the TFP growth rate and A_s is the TFP saturation level.

Capital stock growth occurs through a balance of depreciation and investment from the previous time step,

$$K_t = (1-\delta)K_{t-1} + I_{t-1},$$

where δ is the capital depreciation rate, which is constrained to be less than the savings rate s. Labor input is determined by

$$L_t = \pi P_t,$$

where π is the labor force participation rate. Labor is initialized using the (uncertain) initial population P_0, while capital is initialized using the steady-state relationship

$$K_0 = \left(\frac{sA_0}{\delta}\right)^{1/\lambda} L_0.$$

Carbon Emissions

We model the link between economic output and anthropogenic CO_2 emissions from fossil fuel burning and cement production using a time-dependent carbon intensity of production. That is, we assume that energy is a derived demand, rather than a factor of production. The carbon emissions C_t at time t are

$$C_t = Q_t\phi_t,$$

where ϕ is the carbon intensity. ϕ is modeled as a weighted average of four broadly defined technologies,

$$\phi_t = \sum_{i=1}^{4} \gamma_{i,t}\rho_i,$$

where $\gamma_{i,t}$ is the fraction of the economy invested in technology i, which has a technology-specific carbon intensity ρ_i. This assumes that each fuel is used uniformly across end-uses. We set the carbon intensity of technology 1 to zero to represent pre-industrial economic activity, which had negligible fossil fuel emissions (while human activity did produce CO_2 emissions during this period (Ruddiman, 2003), the primary driver of these emissions was land-use change, which we do not consider). The carbon intensities of the technologies 2 and 3 are estimated from observations with the constraint $\rho_2 \geq \rho_3$. This constraint represents the transition from a higher carbon intensity technology, analogous to coal, to a lower carbon intensity technology, analogous to oil and natural gas. We set the carbon intensity of technology 4 to zero to simulate the penetration of low-carbon technologies such as nuclear and renewables. The time dynamics of γ_i are approximated as logistic penetration curves,

$$\begin{aligned}
\gamma_{1,t} &= 1 - \frac{1}{1+\exp(-\kappa(t-\tau_2))} \\
\gamma_{2,t} &= \frac{1}{1+\exp(-\kappa(t-\tau_2))} - \frac{1}{1+\exp(-\kappa(t-\tau_3))} \\
\gamma_{3,t} &= \frac{1}{1+\exp(-\kappa(t-\tau_3))} - \frac{1}{1+\exp(-\kappa(t-\tau_4))} \\
\gamma_{4,t} &= 1 - \frac{1}{1+\exp(-\kappa(t-\tau_4))},
\end{aligned}$$

where κ is the rate at which technologies penetrate and τ_i is the time at which technology i has penetrated half the market. This type of logistic penetration model can reasonably approximate observed energy substitution dynamics (Marchetti, 1977; Grübler et al., 1999).

S2 Model Calibration

We use Bayesian data assimilation to calibrate the model and estimate parametric and predictive uncertainty. Bayesian statistical methods allow for the fusion of data with prior information via Bayes' theorem (Bayes, 1763), yielding probabilistic parameter estimates and projections. As our model outputs are necessarily greater than zero, we construct our likelihood function using log-scale residuals. The residuals between the model estimates and the observations are modeled using a vector autoregression process of order 1 (VAR(1)). We use this specification due to the auto- and cross-correlations of the residuals when an independent and identically distributed error assumption is made. The lag-1 auto-correlations are 0.60 for population, 0.49 for gross world product, and 0.94 for emissions. The lag-0 cross-correlations range from 0.13 to 0.58. The mathematical specification of the residual structure model and derivation of the corresponding likelihood function is provided in the Section S3.

The fossil fuel resource constraint is used in the evaluation of the likelihood. After running the model with a proposed set of parameters, the output emissions are compared to the constraint. If the total emissions violate the constraint, the likelihood of the parameters is assigned a value of zero. Similarly, if the technology shares of the global economy are outside of the specified penetration constraint windows, the likelihood of the parameters is assigned a likelihood of zero.

We use Markov chain Monte Carlo (MCMC) with the Metropolis-Hastings algorithm (Metropolis et al., 1953; Hastings, 1970) to draw samples from the posterior distribution for each modeling scenario. Four chains are run to facilitate convergence checks. Each chain is initialized at a maximum a posteriori (MAP) estimate for that scenario and is run for two million iterations. A burn-in period of 500,000 iterations is discarded at the start of each chain. Both the chain and burn-in lengths are determined using a combination of visual inspection and the Gelman-Rubin diagnostic (Gelman and Rubin, 1992). Plots of the resulting marginal prior and posterior distributions for the standard scenario are provided in Fig. S8. The pairs plot of the posterior distribution is provided in Fig. S10.

We show how alternate priors for important parameters, described in Table S3, affect the resulting projections in Fig. S11. Posterior distributions for the standard scenario corresponding to the inclusion of the three expert assessments (including none and all) are shown in Fig. S4. The impact of the expert assessments on projections is shown in Fig. S3.

S3 Derivation of VAR(1) Likelihood Function

Let $\mathbf{z}_t = (z_{1t}, z_{2t}, \ldots, z_{mt})^T$ and $\mathbf{M}(\boldsymbol{\theta})_t = (M(\boldsymbol{\theta})_{1t}, M(\boldsymbol{\theta})_{2t}, \ldots, M(\boldsymbol{\theta})_{mt})^T$ be vectors of the observations and model outputs (corresponding to model parameters $\boldsymbol{\theta}$) at time t, respectively. In our case, $m = 3$, corresponding to population, economic output, and CO_2 emissions. We model the difference between $\mathbf{z}_t$ and $\mathbf{M}(\boldsymbol{\theta})_t$ with a vector autoregressive (VAR) component $\mathbf{x}_t$, which allows model errors to be correlated over time.

The model for the observations has the form

$$\mathbf{z}_t = \mathbf{M}(\theta)_t + \mathbf{x}_t + \boldsymbol{\varepsilon}_t$$
$$\mathbf{x}_t = A\mathbf{x}_{t-1} + \mathbf{w}_t,$$

where $\mathbf{w}_t \sim N(\mathbf{0}, W)$ and $\boldsymbol{\varepsilon}_t \sim N(\mathbf{0}, D)$, with D a diagonal matrix of observation errors. The vectors $\mathbf{x}_t$, $\mathbf{w}_t$, and $\boldsymbol{\varepsilon}_t$ have dimension m, defined similarly as $\mathbf{z}_t$ and $\mathbf{M}(\boldsymbol{\theta})_t$. We assume that the observation errors are independent over time with known errors D, and $\mathbf{w}_t$ are the white

noise process of the VAR model. Hence, $\text{Cov}(\boldsymbol{\varepsilon}_t, \boldsymbol{\varepsilon}_s) = 0$ and $\text{Cov}(\mathbf{w}_t, \mathbf{w}_s) = 0$. Further, we assume that the process $\mathbf{x}_t$ is weakly stationary with $E(\mathbf{x}_t) = 0$.

The marginal covariance of $\mathbf{x}_t$ can be derived from solving the following for Σ_x:

$$\begin{aligned}
\Sigma_x &= \text{Cov}(\mathbf{x}_t) \\
&= E[(A\mathbf{x}_{t-1} + \mathbf{w}_t)(A\mathbf{x}_{t-1} + \mathbf{w}_t)'] \\
&= AE(\mathbf{x}_{t-1}\mathbf{x}'_{t-1})A' + E(\mathbf{w}_t\mathbf{w}'_t) \\
&= A\Sigma_x A' + W,
\end{aligned}$$

which gives

$$\begin{aligned}
\text{vec}(\Sigma_x) &= (A \otimes A)\text{vec}(\Sigma_x) + \text{vec}(W) \\
\text{vec}(\Sigma_x) &= (I - A \otimes A)^{-1}\text{vec}(W).
\end{aligned}$$

Conditional on the model outputs, $\mathbf{z}_t \sim N(\mathbf{M}(\theta)_t, \Sigma_z)$, where

$$\begin{aligned}
\Sigma_z &= \text{Cov}(\mathbf{z}_t) \\
&= E[(\mathbf{x}_t + \boldsymbol{\varepsilon}_t)(\mathbf{x}_t + \boldsymbol{\varepsilon}_t)'] \\
&= E(\mathbf{x}_t\mathbf{x}'_t) + E(\boldsymbol{\varepsilon}_t\boldsymbol{\varepsilon}'_t) \\
&= \Sigma_x + D.
\end{aligned}$$

The covariance for any two observations with lag h is

$$\begin{aligned}
\text{Cov}(\mathbf{z}_t, \mathbf{z}_{t-h}) &= A^h\text{Cov}(\mathbf{x}_{t-1}, \mathbf{x}_{t-h}) \\
&= A^h\Sigma_x.
\end{aligned}$$

Hence, the likelihood function for observations $\mathbf{z} = (\mathbf{z}'_1, \ldots, \mathbf{z}'_T)'$ is

$$L(\mathbf{z}; \boldsymbol{\theta}, A, D, W) = |\Sigma|^{-1/2} \exp\left(-\frac{1}{2}\left(\mathbf{z} - M(\boldsymbol{\theta})\right)' \Sigma^{-1} \left(\mathbf{z} - M(\boldsymbol{\theta})\right)\right),$$

with $\mathbf{M}(\boldsymbol{\theta}) = (\mathbf{M}(\boldsymbol{\theta})'_1, \ldots, \mathbf{M}(\boldsymbol{\theta})'_T)'$ and

$$\Sigma = \begin{pmatrix}
\Sigma_x + D & (A\Sigma_x)' & \ldots & (A^{n-1}\Sigma_x)' \\
A\Sigma_x & \Sigma_x + D & \ldots & (A^{n-2}\Sigma_x)' \\
\vdots & \vdots & \ddots & \vdots \\
A^{n-1}\Sigma_x & A^{n-2}\Sigma_x & \ldots & \Sigma_x + D
\end{pmatrix}.$$

S4 Prior Distributions and Sensitivities

Default Priors

We complete the specifications of the Bayesian hierarchical model by assigning prior distributions to all unknown parameters. Supplemental Tables 1 and 2 list the prior distributions for these parameters. Prior distributions are specified by their family (*e.g.* normal or uniform) and a lower and upper bound. Normal-family distributions (including normal and log-normal distributions) are specified using their 2.5% and 97.5% central probability limits, while uniform distributions are specified using their lower and upper bounds.

Table S1: Prior distributions used in calibration for the model parameters. Lower and upper bounds are absolute bounds for uniform distributions and central 95% probability intervals for normal and log-normal distributions.

Parameter	Description	Units	Prior	Lower Bound	Upper Bound	Reference
ψ_1	population growth rate	1/year	normal	0.0001	0.15	this study
ψ_2	half-saturation constant	1000$/(year capita)	uniform	0	50	this study
ψ_3	population carrying capacity	billions	normal	6.9	14.4	Lutz et al. (1997); [a]
P_0	population in 1700	billions	normal	0.3	0.9	Maddison (2003); [b]
λ	elasticity of production with respect to labor	dimensionless	normal	0.6	0.8	Romer (2012)
s	savings rate	dimensionless	normal	0.22	0.26	this study[c]
δ	capital depreciation rate	1/year	uniform	0.01	0.14	Nordhaus (1994); Nadiri and Prucha (1996)
α	total factor productivity growth rate	1/year	normal	0.0007	0.0212	this study
A_s	saturation level of total factor productivity	dimensionless	uniform	5.3	16.11	Nordhaus (1994);[d]
π	labor participation rate	dimensionless	normal	0.62	0.66	this study;[e]
A_0	total factor productivity in 1700	dimensionless	uniform	0	3	this study;[f]
ρ_2	carbon intensity of technology 2	kg carbon/2011US$	normal	0	0.75	this study
ρ_3	carbon intensity of technology 3	kg carbon/2011US$	normal	0	0.75	this study
τ_2	half-saturation year of technology 2	year	uniform	1700	2100	this study
τ_3	half-saturation year of technology 3	year	uniform	1700	2100	this study
τ_4	half-saturation year of technology 4	year	normal[g]	2050	2150	this study
κ	rate of technological penetration	1/year	uniform	0.005	0.2	Grübler (1991)

[a] The lower bound is the peak population in the 2.5% scenario; the upper bound the 2100 population in the 97.5% scenario.

[b] The lower bound is the minimum of the four alternative estimates of Maddison (2003, Table B-1) minus the standard deviation; the upper bound is the maximum plus the standard deviation.

[c] The global average gross savings rate between 1977 and 2017 is 24% with a standard deviation of 1.1% (World Bank, 2018). The range given here is consistent with that distribution.

[d] We use the ratio of the 2005 level to the long-term saturation level with a uniform probability density function of ±50%.

[e] The global average labor force participation rate between 1990 and 2018 is 64% with a standard deviation of 1.4% (World Bank, 2019). The range given here is consistent with that distribution.

[f] The best guess is obtained using $A_0 = Q_0\lambda/(1+\lambda)(\delta/s)^{\lambda/(1+\lambda)}(\pi P_0)^{\lambda^2/(1+\lambda)}$.

[g] This distribution was truncated from below at 2020.

Table S2: Prior distributions used in calibration for the statistical parameters.

Parameter	Description	Units	Prior	Lower Bound	Upper Bound	Reference
a_{ii}	diagonal entries of VAR coefficient matrix A	dimensionless	normal	0	1	this study
$a_{ij}, i \neq j$	off-diagonal entries of VAR coefficient matrix A	dimensionless	normal	-1	1	this study
σ_1	log-scale population innovation variance	(log-billions)^2	log-normal	0	∞	this study[a]
σ_2	log-scale GWP innovation variance	(log-trillions 2011USD\$)^2	log-normal	0	∞	this study[a]
σ_3	log-scale emissions innovation variance	(log-GtC/yr)^2	log-normal	0	∞	this study[a]
ε_1	log-scale population observation error variance	(log-billions)^2	log-normal	0	∞	this study[a]
ε_2	log-scale GWP observation error variance	(log-trillions 2011USD\$)^2	log-normal	0	∞	this study[a]
ε_1	log-scale emissions observation error variance	(log-GtC/yr)^2	log-normal	0	∞	this study[a]

[a] Log-normal distributions have log-scale means of -1 and log-scale standard deviations of 1.

Sensitivity to Priors

Table S3 specifies alternate priors for particular parameters to detect the sensitivity of projections to the choice of priors. These parameters were selected because they were either identified as important by the sensitivity analysis (Fig. 5) or they were not updated by the Bayesian inversion (Fig. S8). The alternate prior specifications were selected to include unbounded prior ranges (when the prior was previously uniform) or fatter tails (when the prior was previously normal). Other priors are kept the same from the standard scenario.

Fig. S10 shows the projections resulting from calibrating the model with these prior distri-

butions. In general, the projections are identical, and the qualitative features of the marginal distribution in 2100 are preserved.

Table S3: Prior distributions used in calibration for the model parameters. Lower and upper bounds are absolute bounds for uniform distributions and central 95% probability limits for normal and log-normal distributions.

Parameter	Description	Units	Prior	Lower Bound	Upper Bound
λ	elasticity of production with respect to labor	dimensionless	log-normal	0.6	0.8
s	savings rate	dimensionless	log-normal	0.22	0.26
A_s	saturation level of total factor productivity	dimensionless	normal	5.3	16.11
π	labor participation rate	dimensionless	log-normal	0.62	0.66

Figure S2 shows the projections resulting from alternate prior distributions for τ_4, the half-saturation year of the zero-carbon technology. The "alternate" prior is a normal distribution (truncated from below at 2020) with a 95% central confidence interval from 2050 to 2250. We also show projections from the delayed zero-carbon prior from the main manuscript.

S5 Sobol' Sensitivity Indices

To compute the Sobol' sensitivity indices (Sobol', 1993, 2001), the model parameter space is sampled using quasi-random Saltelli sampling (Saltelli et al., 2010). To compute the second-order interactive sensitivity indices, $M = 2n(d + 1)$ samples must be generated, where d is the number of uncertain parameters and n is sufficiently large to achieve the desired level of precision. The larger the value of n, the more precise the estimates of the sensitivity indices, but the procedure will be more computationally expensive. We use $n = 1e5$ samples. Confidence intervals for the estimates are computed using a bootstrapping analysis with $1e4$ replicates. We evaluate the convergence of the estimates based on a manual inspection of the confidence interval width. Tables S4 and S5 report statistically significant indices that were above selected thresholds.

Table S4: First- and total-order Sobol' Sensitivity indices for each sampled parameter that accounts for greater than 1% of total variability in cumulative emissions from 2018-2100. The 95% confidence interval of each index is provided in parentheses. Only statistically significant variables greater than 1% are reported. Total-order sensitivities can add up to be greater than 1 due to multiple second-order interactions.

Parameter	Description	Total-Order Index	First-Order Index
ψ_1	population growth rate	0.15 (0.15, 0.16)	–
ψ_2	half-saturation constant	0.17 (0.17, 0.17)	–
ψ_3	population carrying capacity	0.08 (0.08, 0.08)	–
λ	elasticity of production with respect to labor	0.86 (0.86, 0.87)	0.08 (0.07, 0.08)
s	savings rate	0.02 (0.01, 0.02)	–
δ	capital depreciation rate	0.68 (0.68, 0.69)	0.07 (0.07, 0.07)
α	total factor productivity growth rate	0.87 (0.87, 0.88)	–
A_s	saturation level of total factor productivity	0.37 (0.37, 0.38)	0.05 (0.05, 0.06)
A_0	total factor productivity in 1700	0.54 (0.53, 0.54)	–
ρ_2	carbon intensity of technology 2	0.17 (0.17, 0.17)	0.14 (0.14, 0.15)
ρ_3	carbon intensity of technology 3	0.44 (0.44, 0.44)	0.17 (0.16, 0.17)
τ_3	half-saturation year of technology 3	0.18 (0.18, 0.18)	–
τ_4	half-saturation year of technology 4	0.06 (0.06, 0.06)	0.03 (0.02, 0.03)
κ	rate of technological penetration	0.31 (0.31, 0.31)	0.10 (0.09, 0.10)

Table S5: Second-order Sobol' Sensitivity indices for each interaction between sampled parameters that accounts for greater than 10% of total variability in cumulative emissions from 2018-2100. The 95% confidence interval of each index is provided in parentheses. Only statistically significant interactions greater than 10% are reported.

Parameter 1	Parameter 2	Second-Order Index
ψ_1	ψ_2	0.25 (0.24, 0.25)
λ	δ	0.13 (0.12, 0.13)
λ	α	0.58 (0.57, 0.59)
δ	A_0	0.43 (0.43, 0.44)
α	A_0	0.38 (0.38, 0.38)
ρ_2	τ_3	0.14 (0.14, 0.15)
ρ_3	τ_4	0.10 (0.09, 0.11)
ρ_3	κ	0.17 (0.17, 0.18)
τ_3	κ	0.25 (0.24, 0.26)

References

Ausubel JH (1995) Technical progress and climatic change. Energy Policy 23(4):411–416, DOI 10.1016/0301-4215(95)90166-5

Bayes T (1763) An essay towards solving a problem in the doctrine of chance. Philosophical Transactions of the Royal Society of London 53:370–418

Cohen JE (1995) Population growth and earth's human carrying capacity. Science 269(5222):341–346, DOI 10.1126/science.7618100

Gelman A, Rubin DB (1992) Inference from iterative simulation using multiple simulations. Stat Sci 7(4):457–511, DOI 10.1214/ss/1177011136

Grübler A (1991) Diffusion: Long-term patterns and discontinuities. Technol Forecast Soc Change 39(1):159–180, DOI 10.1016/0040-1625(91)90034-D

Grübler A, Nakićenović N, Victor DG (1999) Dynamics of energy technologies and global change. Energy Policy 27(5):247–280, DOI 10.1016/S0301-4215(98)00067-6

Hastings WK (1970) Monte carlo sampling methods using markov chains and their applications. Biometrika 57(1):97–109, DOI 10.2307/2334940

Lutz W, Sanderson W, Scherbov S (1997) Doubling of world population unlikely. Nature 387(6635):803–805, DOI 10.1038/42935

Lutz W, Sanderson W, Scherbov S (2001) The end of world population growth. Nature 412(6846):543–545, DOI 10.1038/35087589

Maddison A (2003) The World Economy. OECD, DOI 10.1787/9789264104143-en

Marchetti C (1977) Primary energy substitution models: On the interaction between energy and society. Technol Forecast Soc Change 10(4):345–356, DOI 10.1016/0040-1625(77)90031-2

Metropolis N, Rosenbluth AW, Rosenbluth MN, Teller AH, Teller E (1953) Equation of state calculations by fast computing machines. J Chem Phys 21(6):1087–1092, DOI 10.1063/1.1699114

Nadiri MI, Prucha IR (1996) Estimation of the depreciation rate of physical and R&D capital in the U.S. total manufacturing sector. Econ Inq 34(1):43–56, DOI 10.1111/j.1465-7295.1996.tb01363.x

Nordhaus WD (1994) Managing the global commons : the economics of climate change. MIT Press, Cambridge, Mass.

Nordhaus WD, Yohe GW (1983) Future carbon dioxide emissions from fossil fuels. In: National Research Council (ed) Changing Climate: Report of the Carbon Dioxide Assessment Committee, The National Academies Press, Washington, DC, pp 87–153, DOI 10.17226/18714

Romer D (2012) Advanced Macroeconomics, fourth ed. edn. McGraw-Hill/Irwin, New York

Ruddiman WF (2003) The anthropogenic greenhouse era began thousands of years ago. Clim Change 61(3):261–293, DOI 10.1023/B:CLIM.0000004577.17928.fa

Saltelli A, Annoni P, Azzini I, Campolongo F, Ratto M, Tarantola S (2010) Variance based sensitivity analysis of model output. design and estimator for the total sensitivity index. Comput Phys Commun 181(2):259–270, DOI 10.1016/j.cpc.2009.09.018

Sobol' IM (1993) Sensitivity estimates for nonlinear mathematical models. Mathematical Modeling and Computational Experiment 1(4):407–414

Sobol' IM (2001) Global sensitivity indices for nonlinear mathematical models and their monte carlo estimates. Math Comput Simul 55(1):271–280, DOI 10.1016/S0378-4754(00)00270-6

Starr C, Rudman R (1973) Parameters of technological growth. Science 182(4110):358–364, DOI 10.1126/science.182.4110.358

World Bank (2018) Gross savings (% of GDP). https://data.worldbank.org/indicator/ny.gns.ictr.zs?end=2017&start=1960&view=chart, accessed: 2019-5-15

World Bank (2019) Labor force participation rate, total (% of total population ages 15+). https://data.worldbank.org/indicator/sl.tlf.cact.zs, accessed: 2019-5-15

Supplemental Figures

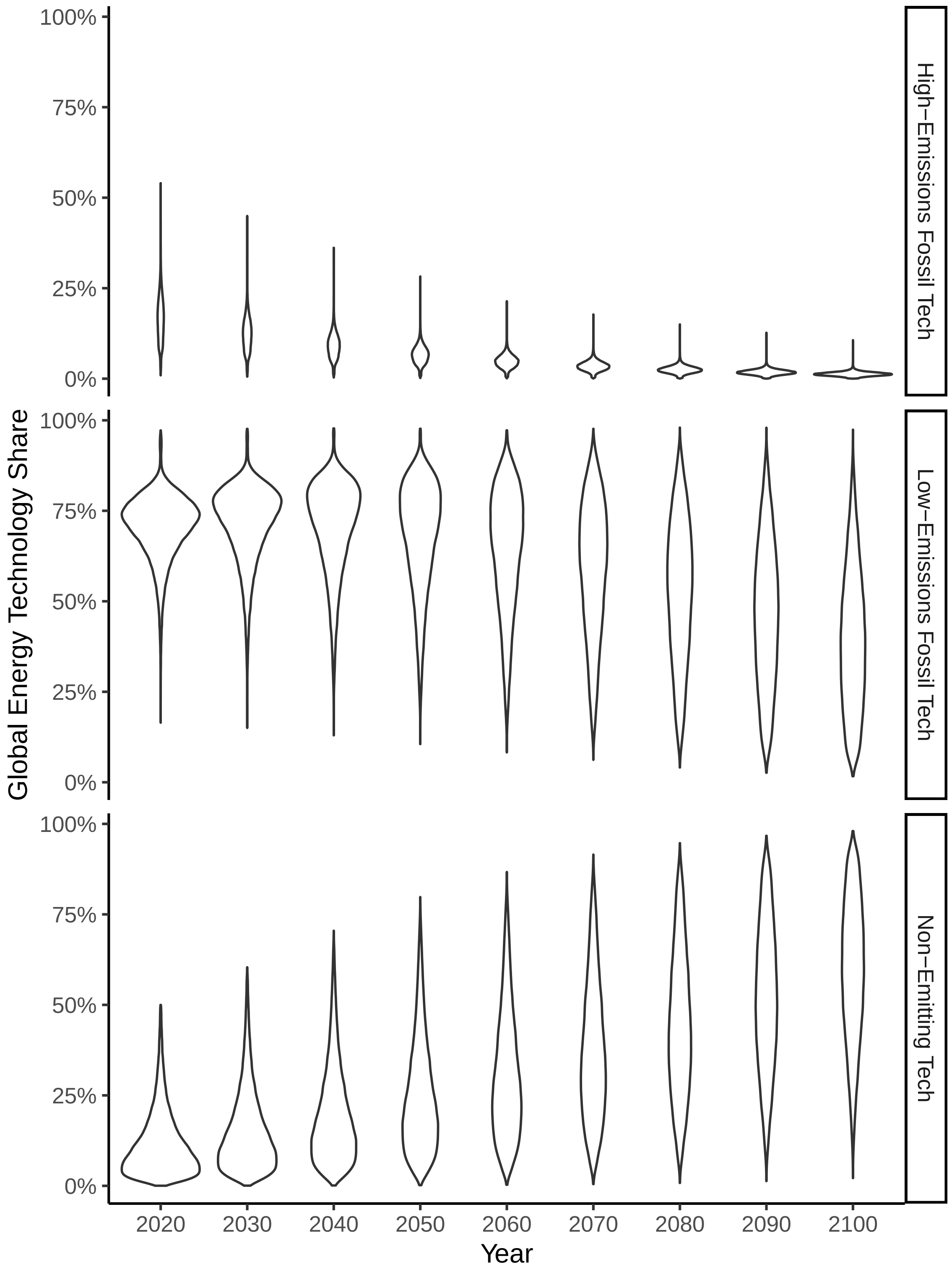

Figure S1: **Technology shares without a penetration constraint** – Violin plots of the shares of the various emitting technologies in our model from 2020–2100 without an active technology penetration constraint in 2019.

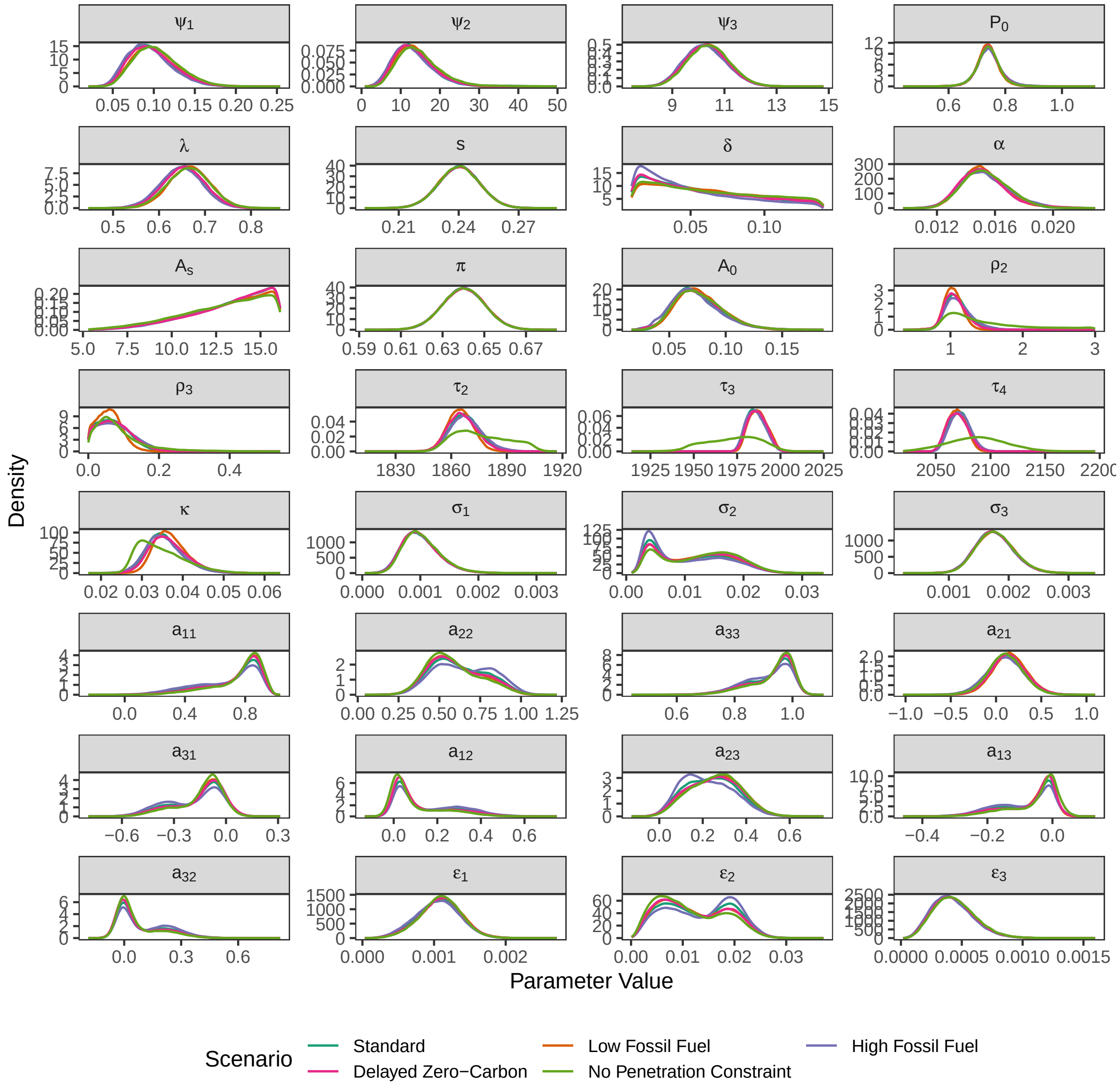

Figure S2: **Parameter posterior distributions by model scenario** – Marginal posterior distributions for the model parameters under each model scenario.

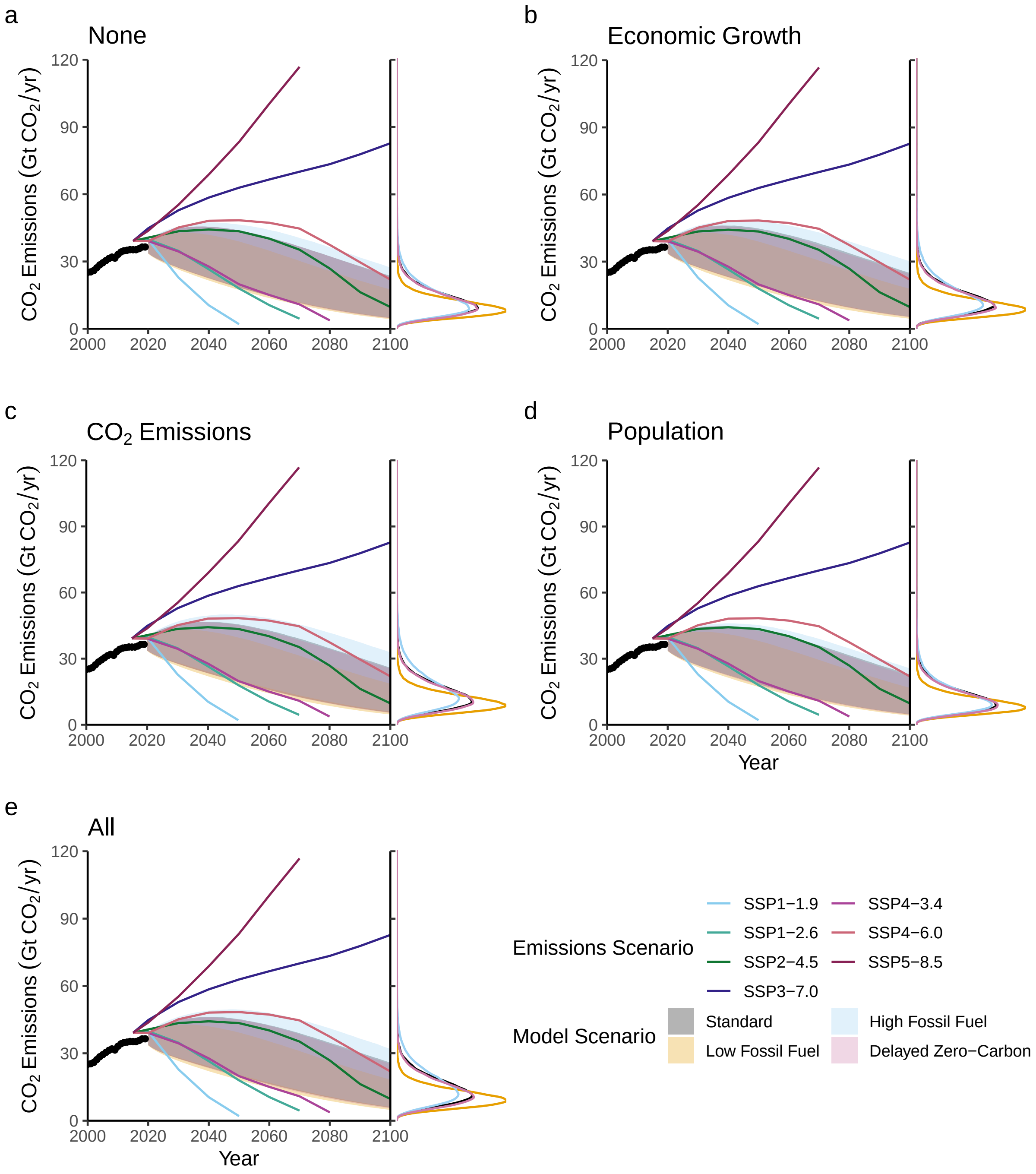

Figure S3: **Sensitivity of carbon dioxide emissions projections to expert assessments** – Time series and marginal distribution in 2100 for annual CO_2 emissions projections with respect to varying expert assessment assimilations: a) No expert assessments; b) only Christensen et al (2018)[2]1; c) only Ho et al (2019)[8]; d) only United Nations (2019); e) all three.

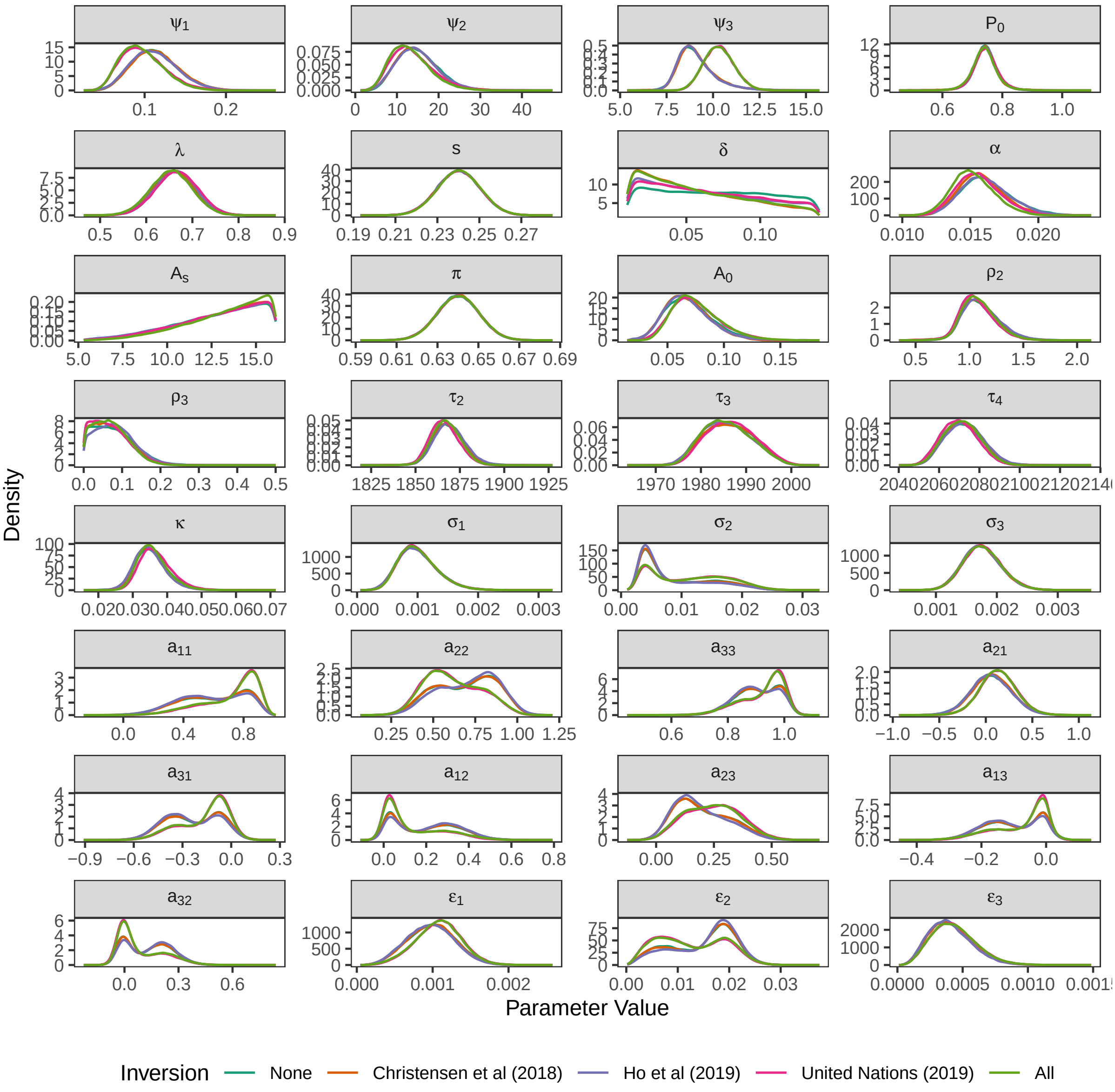

Figure S4: **Expert assessment impact on model parameter posterior distributions** – Posterior distributions for the model parameters under the standard scenario with respect to calibrations assimilating different expert assessments.

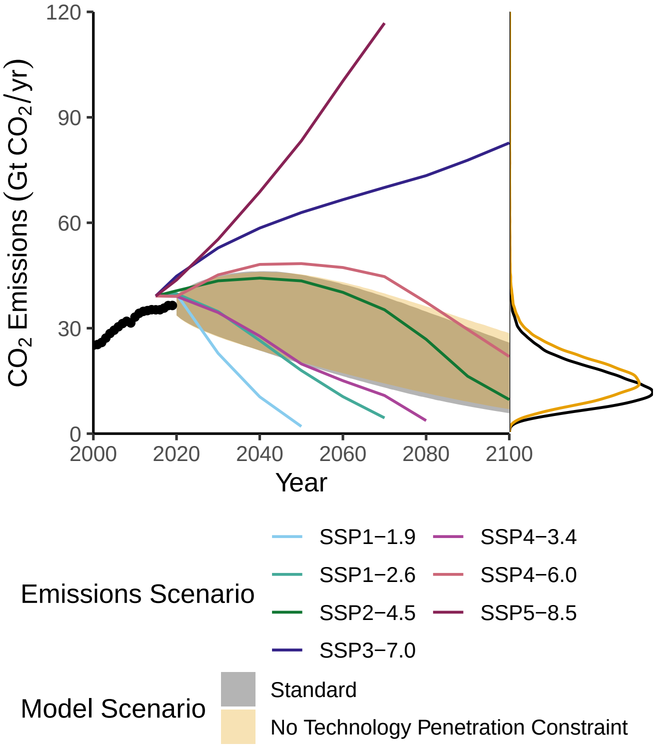

Figure S5: **Impact of technology penetration constraint on projected emissions** – Time series and marginal distribution in 2100 for annual CO_2 emissions under the standard scenario assumptions, with and without the technology penetration constraint.

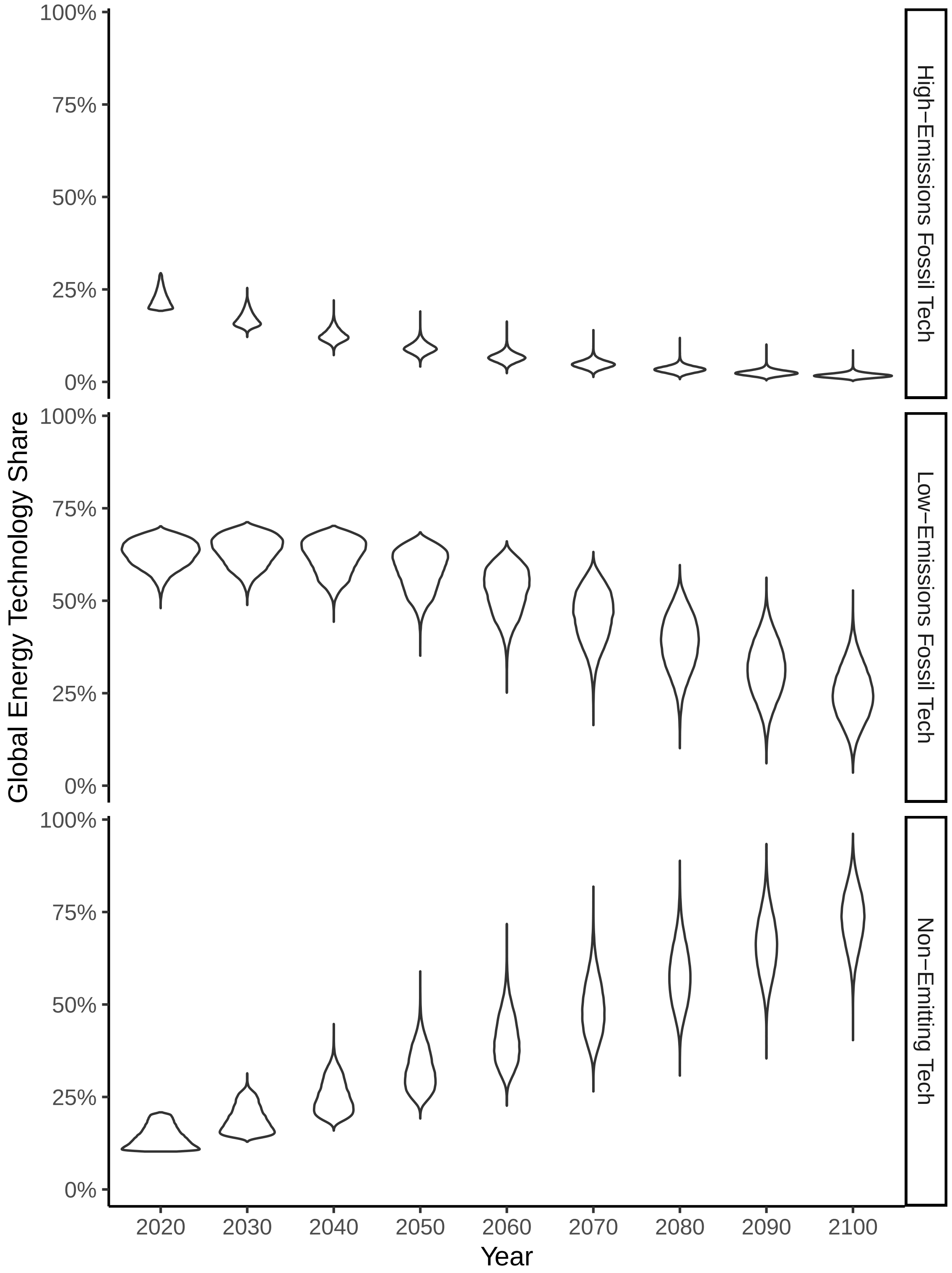

Figure S6: **Technology shares with the penetration constraint** – Violin plots of the shares of the various emitting technologies in our model from 2020–2100 with our technological constraint.

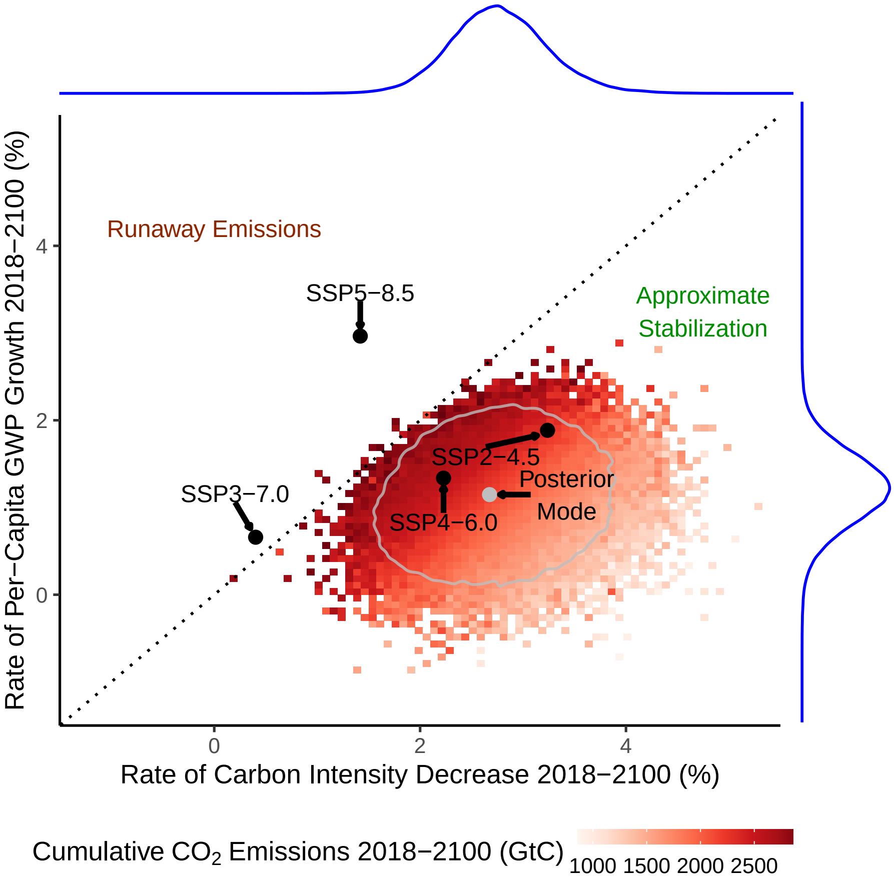

Figure S7: **Cumulative emissions from 2018-2100 by rates of global economic growth and carbon intensity decrease** – Mean cumulative emissions from 2018-2100 are shown with respect to each binned region of average annual rates of global economic growth and carbon intensity decrease. Relevant SSP-RCP scenarios are shown as black dots. The grey contour is the 95% posterior region and the posterior mode is represented by a grey dot. The dotted diagonal line is the 1-1 line corresponding to equal rates of economic growth and carbon intensity decrease. Regions corresponding to runaway emissions growth and approximate stabilization are labeled. The marginal distributions of rates of global economic growth and carbon intensity decrease are shown in blue.

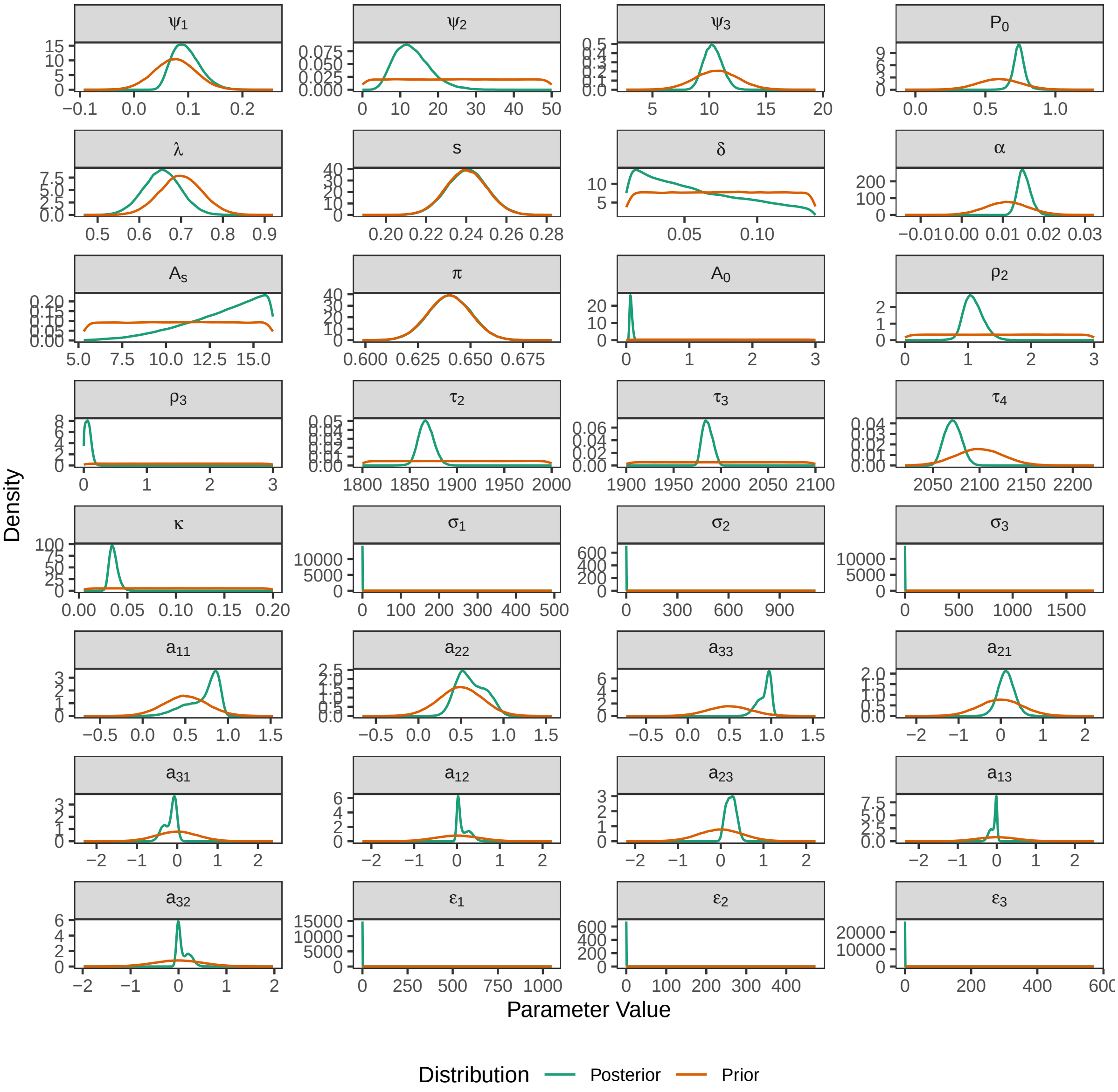

Figure S8: **Prior and posterior distributions for model parameters** – Prior and posterior distributions for the model parameters under the standard scenario. Prior distributions are in orange; posterior distributions are in green.

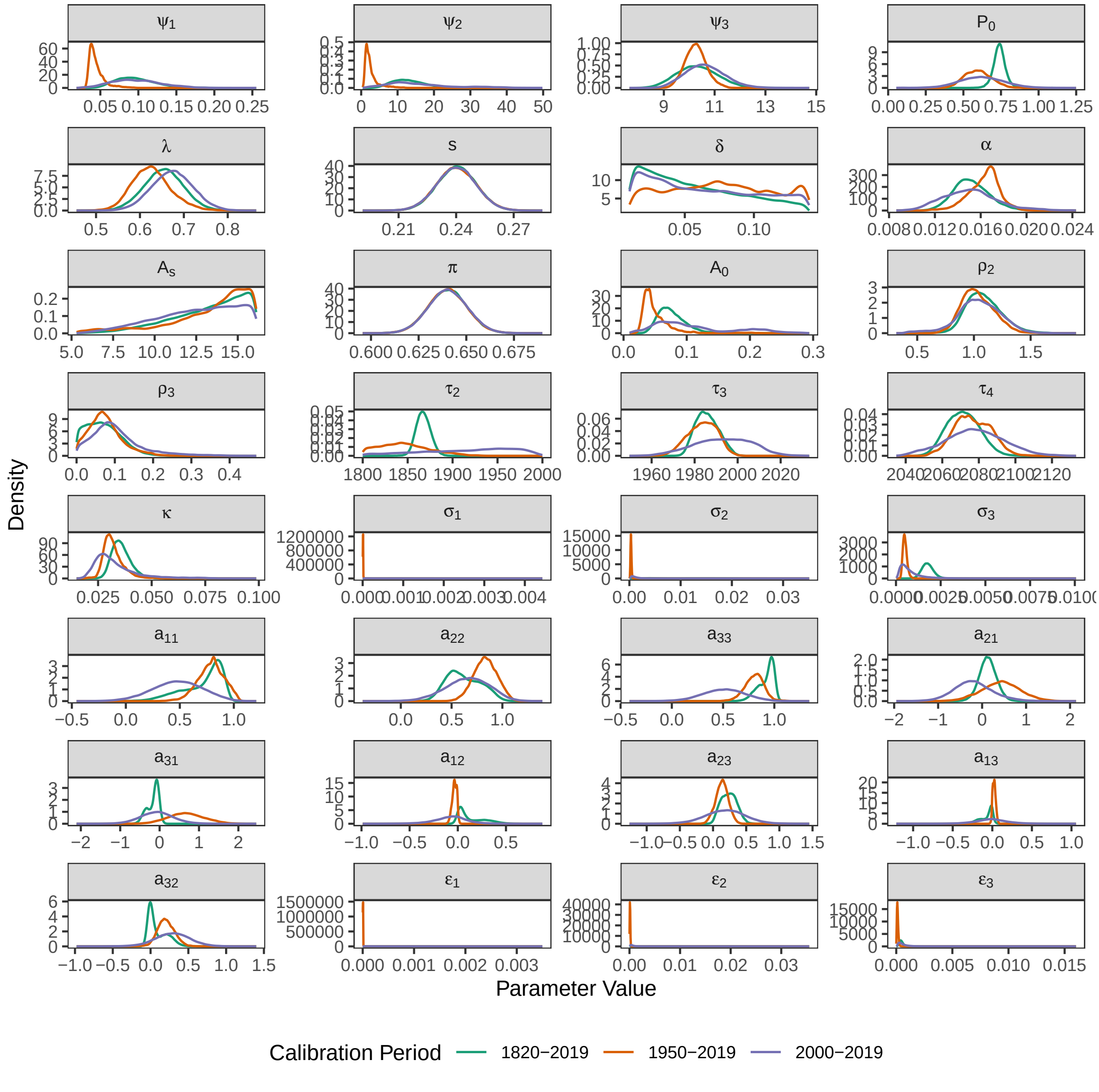

Figure S9: **Parameter posterior distributions by model scenario** – Marginal posterior distributions for the model parameters under each model scenario.

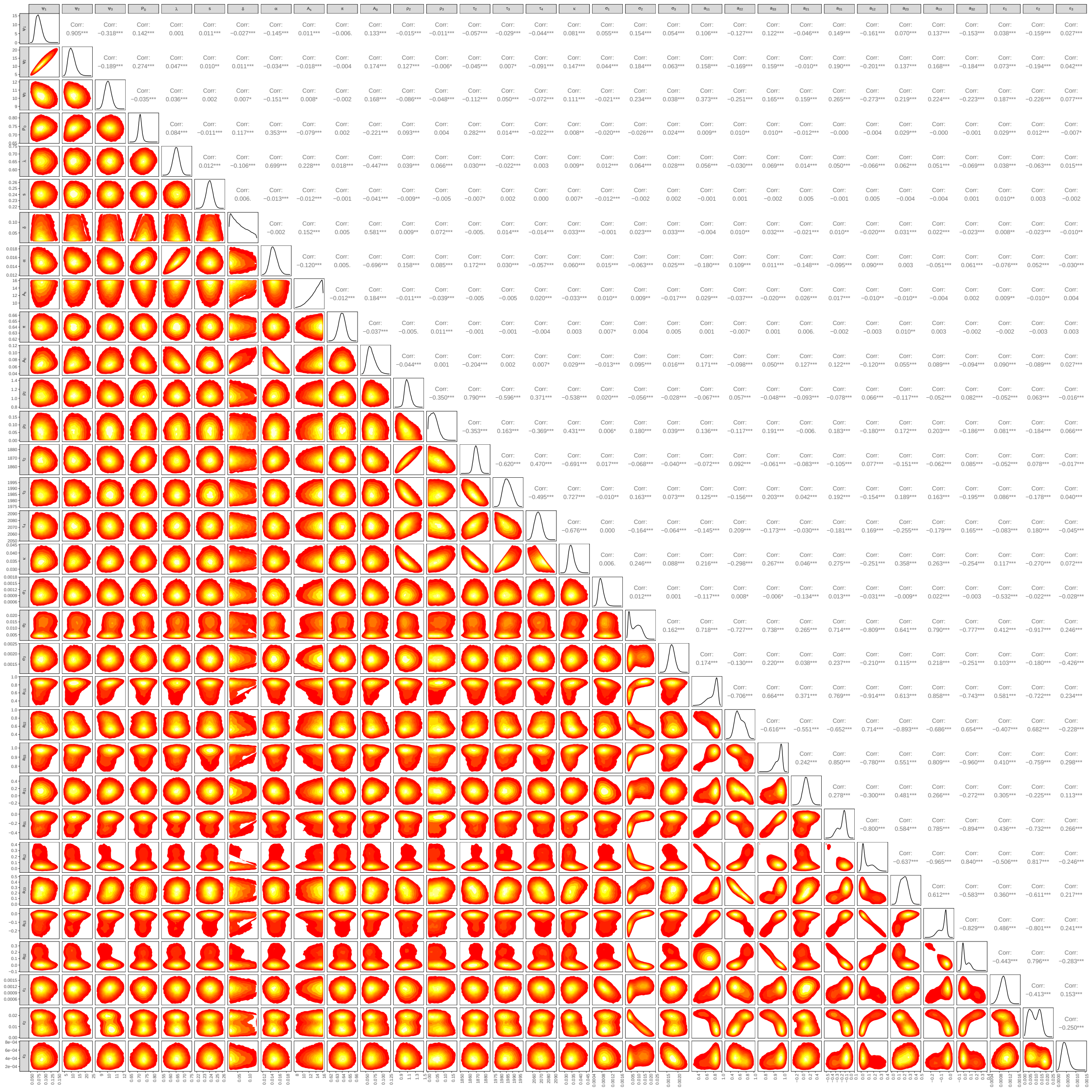

Figure S10: **Pairs plot for the standard calibration** – Pairs plot of the posterior distribution resulting from the standard calibration. Marginal distributions are plotted along the diagonal. The elements below the diagonal are heatmaps showing the joint bivariate densities. Correlation coefficients between pairs of parameters are provided above the diagonal.

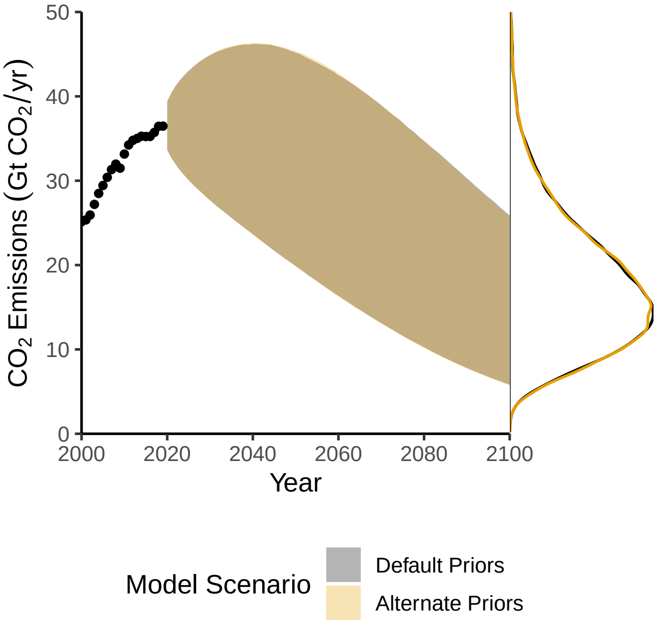

Figure S11: **Sensitivity of CO_2 emissions projections to prior distributions** – Projections are for the default priors (Supplemental Table 1 and Supplemental Table 2) and an alternate set of priors (Supplemental Table 3). The shaded regions are the 90% credible intervals. Black dots are observations. The marginal distribution of projected business-as-usual CO_2 emissions in 2100 is shown on the right.
